# *Snca*-GFP Knock-In Mice Reflect Patterns of Endogenous Expression and Pathological Seeding

**DOI:** 10.1523/ENEURO.0007-20.2020

**Published:** 2020-08-27

**Authors:** Anna Caputo, Yuling Liang, Tobias D. Raabe, Angela Lo, Mian Horvath, Bin Zhang, Hannah J. Brown, Anna Stieber, Kelvin C. Luk

**Affiliations:** 1 Department of Pathology and Laboratory Medicine, Center for Neurodegenerative Disease Research, University of Pennsylvania Perelman School of Medicine, Philadelphia, PA 19104-4283; 2 Department of Medicine, Division of Translational Medicine and Human Genetics, University of Pennsylvania Perelman School of Medicine, Philadelphia, PA 19104-4283

**Keywords:** α-synuclein, green fluorescent protein, knock-in mouse, Lewy pathology, preformed fibrils

## Abstract

α-Synuclein (aSyn) participates in synaptic vesicle trafficking and synaptic transmission but its misfolding is also strongly implicated in Parkinson’s disease (PD) and other neurodegenerative synucleinopathies in which misfolded aSyn accumulates in different regions of the central and peripheral nervous systems. Although increased aSyn expression levels or altered aggregation propensities likely underlie familial PD with *SNCA* amplification or mutations, the majority of synucleinopathies arise sporadically, indicating that disease can develop under normal levels of wild-type (wt) aSyn. We report here the development and characterization of a mouse line expressing an aSyn-green fluorescence protein (GFP) fusion protein under the control of native *Snca* regulatory elements. Regional and subcellular localization of the aSyn-GFP fusion protein in brains and peripheral tissues of knock-in (KI) mice are indistinguishable from that of wt littermates. Importantly, similar to wt aSyn, aSyn-GFP disperses from synaptic vesicles on membrane depolarization, indicating that the tag does not alter normal aSyn dynamics at synapses. In addition, intracerebral injection of aSyn pre-formed fibrils into KI mice induced the formation of aSyn-GFP inclusions with a distribution pattern similar to that observed in wt mice, albeit with attenuated kinetics because of the GFP-tag. We anticipate that this new mouse model will facilitate *in vitro* and *in vivo* studies requiring *in situ* detection of endogenous aSyn, thereby providing new insights into aSyn function in health and disease.

## Significance Statement

α-Synuclein (aSyn) participates in synaptic vesicle function and represents a major component of the Lewy pathology found in Parkinson’s disease (PD) and related neurodegenerative diseases. The function of aSyn and the sequence of events leading to its aggregation and neurotoxicity are not fully understood. Here, we present a new mouse model in which enhanced green fluorescence protein (GFP) was knocked in at the C terminus of the *Snca* gene. The resulting fusion protein shows identical expression and localization to that of wild-type (wt) animals, is functional, and is incorporated into pathologic aggregates *in vitro* and *in vivo*. This new tool allows for monitoring aSyn under a variety of physiological and pathologic conditions, and may uncover additional insights into its function and dysfunction.

## Introduction

α-Synuclein (aSyn) is a protein prominently expressed in neurons and enriched at presynapses (Maroteaux et al., 1988). Although its precise functions are not fully understood, collective evidence suggests that aSyn regulates synaptic vesicle trafficking and fusion (Nemani et al., 2010; Wang et al., 2014; Logan et al., 2017; Burré et al., 2018). aSyn also constitutes the major component of Lewy bodies and Lewy neurites, intraneuronal inclusions characteristic of Parkinson’s disease (PD) and a group of neurodegenerative diseases known as synucleinopathies (Burré et al., 2018; Bendor et al., 2013; Goedert et al., 2013). While mutations or amplification of the aSyn gene can lead to familial PD (Polymeropoulos et al., 1997; Krüger et al., 1998; Singleton et al., 2003; Zarranz et al., 2004; Koros et al., 2017; Schneider and Alcalay, 2017), the majority of PD cases are sporadic and develop in the presence of normal levels of wild-type (wt) aSyn.

Several aSyn animal models have been generated to study aSyn function and pathobiology (Visanji et al., 2016). Accumulation of aggregated aSyn, loss of dopaminergic neurons, and behavioral impairments have been reported in multiple lines. However, the majority of these models rely on the overexpression of human aSyn, often bearing disease-associated mutations, under the control of a heterologous (i.e., non-*Snca*) promoter, and in the presence of endogenous mouse aSyn expression (Masliah et al., 2000; van der Putten et al., 2000; Kahle et al., 2001; Matsuoka et al., 2001; Giasson et al., 2002; Tofaris et al., 2006; Emmer et al., 2011). These features potentially confound data interpretation, especially in relation to normal aSyn function and its role in sporadic PD. These models also poorly recapitulate the prion-like propagation of pathologic aSyn suggested by the staged distribution of aSyn deposits in diseased human brains (Braak et al., 2003; Alafuzoff et al., 2009; Beach et al., 2009). Recent studies demonstrating that aSyn pathology formation and spread can be initiated in wt mice following inoculation of recombinant aSyn preformed fibrils (PFFs; Luk et al., 2012; Masuda-Suzukake et al., 2014; Rey et al., 2018) or human brain derived aSyn aggregates (Masuda-Suzukake et al., 2013; Recasens et al., 2014; Peng et al., 2018) further support this hypothesis, thus enabling the modeling of these processes without aSyn ectopic expression. Exposure to PFFs also induces aSyn pathology in primary neurons, allowing for cellular and molecular characterizations (Volpicelli-Daley et al., 2011). These models provide additional structural and temporal resolution for observing aSyn pathogenesis, yet they do not allow monitoring aSyn in real time. To this aim, two mouse models overexpressing human aSyn tagged with green fluorescence protein (GFP) have been reported (Rockenstein et al., 2005; Hansen et al., 2013). Transient overexpression of aSyn-GFP has also been used in primary neurons (McLean et al., 2001; Fortin et al., 2005; Volpicelli-Daley et al., 2014a). These models have provided valuable insights about the trafficking of aSyn at synapses (Fortin et al., 2005), aSyn aggregate formation and development (Unni et al., 2010; Osterberg et al., 2015), and their link to synaptic/axonal dysfunction (Scott et al., 2010; Volpicelli-Daley et al., 2014a) and cell death (Osterberg et al., 2015).

Nonetheless, aSyn is differentially expressed in specific neuron subtypes (Hawrylycz et al., 2012; Uhlén et al., 2015; Taguchi et al., 2016), and its function and propensity to aggregate are dependent on expression levels (Nemani et al., 2010; Scott and Roy, 2012; Logan et al., 2017; Luna et al., 2018), thus making the use of the endogenous promoter highly desirable. We therefore generated a mouse in which GFP was knocked in at the C terminus of the endogenous *Snca* gene. The resulting mouse displays aSyn-GFP expression at wt levels, primarily in neurons and with the same distribution as wt aSyn. Moreover, the fusion-protein localizes correctly to synaptic vesicle and participates in the synaptic vesicle cycle. Importantly, aSyn-GFP is incorporated in Lewy-like pathology seeded by exposure to PFFs. We anticipate that this new tool will allow for further studies aimed at better understanding aSyn physiology and pathobiology.

## Materials and Methods

### Animals

**Figure 1.**
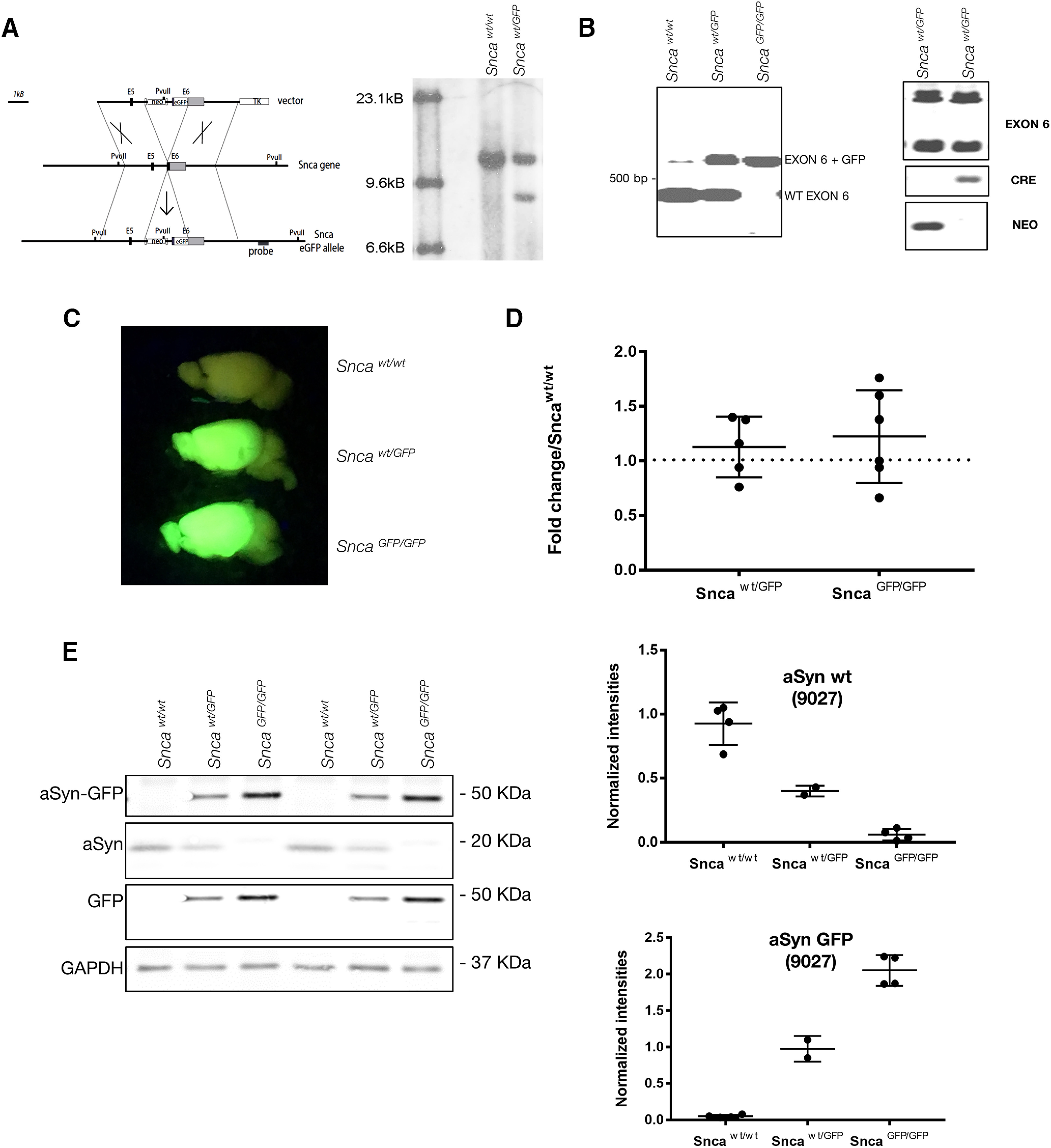
Design and validation of *Snca*-GFP KI mouse. ***A***, The GFP sequence was knocked in (KI) at the C-terminal end (exon 6; E6) of the *Snca* gene, resulting in the expression of aSyn-GFP under the endogenous regulatory elements (for additional details, see Materials and Methods). Right panel, Southern blot analysis of geneticin-resistant ES cells electroporated with the targeting vector in ***A*** following enzymatic digestion with the restriction enzyme PvuII. Note the additional PvuII restriction site introduced by the integration of the targeting vector. ***B***, PCR amplification of wt and *Snca*-GFP exon 6 from heterozygous and homozygous mice. Exon 6 comprises the fusion region. ***C***, Brains from heterozygous and homozygous *Snca*-GFP mice fluoresce when illuminated under blue light. ***D***, Quantification of aSyn mRNA levels in brain homogenates of heterozygous or homozygous KI mice compared with wt littermates. Data are expressed as fold change of aSyn normalized to actin. Mean and SD values are shown (*N* = 5–6 per condition). No statistically significant difference was found between groups using one-way ANOVA (Kruskal–Wallis test and Dunn’s multiple comparison test). ***E***, Levels of aSyn and aSyn-GFP in brain homogenate from wt, heterozygous, and homozygous mice. Graphs show quantification of the immunoblot data expressed as intensities of aSyn or aSyn-GFP (detected using Syn9027) normalized to GAPDH levels. Complete blot and standards are shown in Extended Data Figures 1-1, 1-2, 1-3. Mean and SEM values shown (*N* = 2–4 per group).

All housing, breeding, and procedures were performed according to the *NIH Guide for the Care and Use of Experimental Animals* and approved by the University of Pennsylvania Institutional Animal Care and Use Committee. Animals were anesthetized with a mix of 100 mg/kg ketamine, 10 mg/kg xylazine, and 0.5 mg/kg acepromazine before performing transcardiac perfusion with PBS + heparin (2 USP/ml). The *Snca*-GFP KI line was created by homologous recombination: a synthetic mouse *Snca* exon 6 with a short linker (the primary sequence around the linker being …EPEA-KL-MVSKG…) was inserted directly after the last amino acid of the mouse *Snca* coding sequence, followed by the enhanced GFP coding sequence and the remainder of the *SNCA* 3’UTR (Fig. 1*A*). A synthetic DNA construct (Blueheron Inc.) consisting of ∼200 nt of the genomic *Snca* sequences upstream of exon 6 and all of exon 6 (with the above linker and eGFP sequence inserted directly after the C terminus of *Snca*) was cloned directly downstream of the floxed Neomycine (Neo) cassette of PL452 (NCI). A ∼500-nt-long *Snca* upstream arm of homology was added upstream of the Neo cassette. This construct was introduced by BAC recombineering into pL253 (NCI) harboring ∼12 kB of the genomic *SNCA* region containing at its center *SNCA* exon 6. The resulting targeting vector contained a 14,183-nt-long *Snca* allele with integrated floxed Neo cassette as well as *Snca* exon 6 containing KL–eGFP downstream of the *Snca* protein. The targeting vector was linearized with NotI and introduced into V6.5 ES cells by electroporation. After geneticin selection ES clones were screened by Southern blotting (HindIII digestion) and five positive clones were found and further validated by a second round of Southern blotting (PvuII digestion) using a different probe (Fig. 1*A*). The positive clones generated a wt ∼12 kB and a mutant ∼9kB PvuII fragment. Positive clones were subjected to chromosome counting. Clone 2B9, which had the correct number of chromosomes, was injected into C57/B6 mouse blastocysts. Heterozygous and homozygous mice were obtained and confirmed by PCR (Table 1) using primers encompassing the fusion region (exon 6) that amplify a longer fragment when the GFP sequence is present (Fig. 1*A*,*B*) and resulted in phenotypically normal mice until 24 months of age. No effect on survival was observed (data not shown). *Snca*-GFP mice were bred with a Cre-recombinase expressing line (The Jackson Laboratory stock 006054) to remove the Neo cassette. Animals negative for the Neo gene were identified by PCR (Table 1) and selected for subsequent breeding (Fig. 1*B*), although no differences were observed between Neo positive and Neo negative or mice. C57BL/6J were purchased from The Jackson Laboratory (JAX 000664) and CD1 mice from Charles River (Strain 022). *Snca*^−/−^ mice (Abeliovich et al., 2000) were maintained on a B6C3H background. *Snca*-GFP mice will be made available through The Jackson Laboratory Repository (Stock No. 035412).

### Antibodies

All antibodies used in this study are outlined in Table 2.

**Table 1:** Primer, Sequence, Use

Cre forward	5′ gtaggtggaaattctagcatcatcc 3’	PCR, genotyping
Cre reverse	5’ ctaggccacagaattgaaagatct 3’	PCR, genotyping
Neomycin cassette forward	5’ cggtgccctgaatgaactgc 3’	PCR, genotyping
Neomycin cassette reverse	5’ gatactttctcggcaggagcaa 3’	PCR, genotyping
Exon 6 forward	5’ ggctaccaagactatgagcctg 3’	PCR, genotyping
Exon 6 reverse	5’ gccgatcactgctgtgatggaag 3’	PCR, genotyping
aSyn3 forward	5’ aatgttggaggagcagtggtgact 3’	RT-PCR
aSyn3 reverse	5’ atccacaggcatgtcttccaggat 3’	RT-PCR

**Table 2:** Antibodies used in this study

Antibody	Antigen	Host	Use and dilution	Source
9027	aSyn	Mouse	WB 1:10,000 IF 1:5000	CNDR (Covell et al., 2017)
SNL-1	aSyn	Rabbit	WB, IF 1:1000	CNDR (Giasson et al., 2002)
GFP	Green fluorescence protein	Rabbit	WB 1:1000	Novus Biologicals
GFP	Green fluorescence protein	Chicken	IF 1:2000	Aves labs
GFAP	Glial fibrillary acidic protein	Rabbit	IF 1:1000	DAKO
MAP-2 (17028)	Microtubule-associated protein 2	Rabbit	IF 1:2000	CNDR (Volpicelli-Daley et al., 2011)
Iba-1	Macrophage/microglia-specific calcium-binding protein (Iba1)	Rabbit	IF 1:2000	DAKO
GAPDH (6C5)	Glyceraldehyde-3-phosphate dehydrogenase	Mouse	WB 1:15,000	Advanced Immunochemical
Synapsin 1 AB1543	Synapsin 1 a and b	Rabbit	IF 1:1000	Millipore
Tuj-1	Neuronal Class III β-tubulin (TUJ1)	Mouse	IF 1:1000	BioLegend
vGlut 135304	VGLUT 1 (vesicular glutamate transporter 1)	Guinea Pig	IF 1:2000	Synaptic Systems
81A	a-synuclein phospho S129	Mouse	IF, IHC 1:8000	CNDR (Waxman et al., 2008)
MJFR13	a-synuclein phospho S129	Rabbit	WB 1:1000	Abcam

WB, Western blotting.

IF, immunofluorescence.

IHC, immunohystochemistry.

### Reagents and chemicals

All reagents were purchased from ThermoFisher and chemicals purchased from Sigma unless otherwise indicated.

### Recombinant aSyn monomers and PFFs

Recombinant mouse aSyn and aSyn-GFP were produced as previously described (Luk et al., 2012; Karpowicz et al., 2017) . In brief, plasmids encoding the two proteins were introduced in *Escherichia coli* using heat shock and proteins were purified following lysis using size exclusion and anion exchange chromatography. Purified protein (monomer) was concentrated to 5 mg/ml for aSyn wt and 15 mg/ml for aSyn-GFP (360 μm) and kept frozen until use. Recombinant PFFs (mouse wt aSyn) were produced by shaking 500 μl of recombinant aSyn monomer (5 mg/ml, 360 μm) at 37° C for 7 d. Fibril formation was confirmed by sedimentation assay (see Sedimentation assay section). Right before their use, PFFs are diluted in PBS and sonicated for 10 cycles (30 s ON, 30 s OFF, high intensity) in a bath sonicator at 10°C (BioRuptor; Diagenode).

### Electron microscopy

Wt aSyn (2.5 mg/ml, 180 μm), aSyn-GFP (7.5 mg/ml, 180 μm), or wt aSyn + aSyn-GFP (assembled as 1:1 monomer mixture) were diluted in PBS, absorbed onto Formvar/carbon film-coated copper grids (Electron Microscopy Sciences), washed twice with water, and negatively stained with 0.4–2% uranyl acetate. Grids were visualized with a Jeol 1010 transmission electron microscope (Peabody).

### Sedimentation assay

Monomers were thawed and ultracentrifuged at 100,000 × *g* for 30 min at room temperature (RT). They were then diluted to 2.5 (wt aSyn, 180 μm) and 7.5 mg/ml (aSyn-GFP, 180 μm) in PBS; 100 μl of aSyn, aSyn-GFP, or aSyn + aSyn-GFP (1:1) was aliquoted in Eppendorf tubes and shaken at 1000 rpm at 37°C for 24 h; 6 μl of each reaction was collected at the indicated time points, diluted in 60 μl of PBS, and layered on a 25% sucrose cushion before undergoing ultracentrifugation (30 min at 100,000 × *g*). Supernatant and pellet fractions were separated. Pellets were resuspended in 120 μl of 12.5% sucrose; 25 μl of 5× SDS sample buffer were added to each tube, and 30 μl was loaded on 15% acrylamide gels for SDS-PAGE. Separated proteins were then stained with Coomassie Blue ON and destained with 10% isopropanol, 10% acetic acid, and water before image acquisition with an infrared scanner (LiCor Odyssey).

### Sequential extraction

Neurons were washed twice with PBS and lysed in TBS (50 mm TRIS and 150 mm NaCl; pH 7.6) + 1% Triton X-100 (TX-100). Lysates were sonicated (10 cycles, 1 s ON, 30 s OFF, medium intensity in a bath sonicator at 10°C (BioRuptor; Diagenode) and then rotated at 4°C for 20 min. Lysates were then spun at 100,000 × *g* for 30 min at 4°C, supernatants were removed and labeled as TX-100 soluble fraction. The pellet was washed once with 1% TX-100 TBS, sonicated, and ultracentrifuged. Supernatants were discarded and pellets dissolved in 1/10 volume of TBS, 2% SDS, sonicated, and ultracentrifuged. Supernatants were collected as SDS soluble fraction and pellets were discarded. Protein content was measured by BCA in the TX-100 soluble fraction. Equal amount of total proteins from TX-100 soluble fractions were separated by SDS-PAGE (4–20% gradient gel). An equal volume of the SDS soluble fraction was loaded alongside. Proteins were detected by Western blotting with the indicated antibodies.

### Immunofluorescence

#### Primary neurons

Neurons were fixed at the indicated Days *in vitro* (DIV) with warm 4% paraformaldehyde containing 4% sucrose with or without 1% TX-100 for 15 min at RT. Cells were permeabilized and non-specific binding sites blocked using PBS containing 0.1% TX-100, 3% BSA, 3% fetal bovine serum (FBS) for 20 min. Primary antibodies were added to cells for 1 h, followed by three washes with PBS and 1-h incubation with the appropriate secondary antibody (1:1000). Cells were washed three times with PBS, once in water and mounted using Fluoromount-G.

#### Forty-micrometer brain sections and intestinal whole mounts

Tissues (brain and intestines) were post-fixed in 4% paraformaldheyde. Brain sections were cut using a compresstome (Precisionary) and intestine samples were opened along the mesenteric border and pinned down before fixation. Sections were permeabilized and blocked in PBS containing 10% FBS, 3% BSA, 0.5% TX-100 for 1 h at RT, incubated with primary antibodies ON at RT, washed three times with PBS, incubated with secondary antibodies for 2 h at RT, washed, and mounted using Fluoromount-G (brain sections) or 1:1 PBS/glycerol (intestine).

#### Six-micrometer sections

Sections were produced and treated as described in the immuonohystochemistry section with the modification that after the ON incubation with primary antibodies, sections were incubated with a fluorescently labeled secondary antibody for 2 h at RT, and mounted using fluoromount-G.

Images were captured on a Nikon Ds-Qi1Mc digital camera attached to a Nikon Eclipse Ni microscope (6-µm sections), a Leica Confocal SP8 for colocalization studies (primary neurons and 40-µm sections), a PerkinElmer Lamina Scanner (full 40-µm sections), or using the InCell Analyzer 2200 (GE Healthcare) when using 96-well plates). Analysis was performed using Fiji or Developer software (GE Healthcare, 96-well plates).

### Immunohistochemistry

PBS-perfused mouse brains were post fixed in ethanol (70% in 150 mm NaCl) ON at 4°C, cut in 3-mm slabs, and embedded in paraffin. Tissue was then sectioned at 6 µm using a microtome and applied on glass slides. Before staining, sections were de-paraffinized, rehydrated (xylene, 95%, 90%, and 75% ethanol) and treated with 5% hydrogen peroxide (in methanol). Sections were blocked in TBS containing 3% FBS and 2% BSA for 1 h at RT, incubated with primary antibodies at 4°C ON, with biotinylated secondary antibodies for 1 h at RT (Vector Laboratories), horse radish peroxidase-conjugated streptavidin for 1 h at RT (Vector Laboratories), and signal was revealed using DAB peroxidase substrate products (dark brown, Vector Laboratories). Sections were counterstained with hematoxylin for 1 min and mounted using Cytoseal Mounting Media. Images were captured on a Nikon Ds-Qi1Mc digital camera attached to a Nikon Eclipse Ni microscope or using a Lamina Scanner (PerkinElmer; 20× objective).

### Synaptic vesicle cycling

Hippocampal neurons were plated on MatTek dishes and kept in culture for 18–21 d. Cells were then incubated in Krebs Ringer HEPES buffer (5 mm KCl, 140 mm NaCl, 10 mm HEPES, 10 mm glucose, 2.6 mm CaCl_2_, and 1.3 mm MgCl_2_) for 15 min before starting the imaging sessions. Images were acquired with a Leica microscope and a 40× air objective. Cells were imaged (every 3–5 s) in KRH solution for 2–3 min to establish a baseline signal. The media was then switched to high potassium (HK; 90 mm KCl, 55 mm NaCl, 10 mm HEPES, 10 mm glucose, 2.6 mm CaCl_2_, and 1.3 mm MgCl_2_) or KRH solution for 2 min. Individual vesicles were fragmented using ImageJ/FIJI and intensities before and after stimulation were determined as ratios after subtracting background signal and adjusting for image drift (Lazarenko et al., 2018).

For fixed samples, cells on coverslips were washed and incubated in KHR for 15 min at RT and then either fixed immediately before treatment, incubated with HK for 2 min before fixation (HK), or washed for an additional 15 min using HRK before fixation (HK + 15-min recovery). Cells were then processed for immunostaining as described above.

### Western blotting

Cells were lysed in lyses buffer (0.5% TX-100, 0.5% deoxycholic acid, 10 mm TRIS, and 100 mm NaCl; pH 8.0) with phosphatase and protease inhibitors. Nuclei and debris were removed by centrifugation (5 min at 1000 × *g*). Total protein content was determined by BCA, and equal amounts of total protein were separated on 4–20% gradient gels. Proteins were then transferred to 0.22-µm nitrocellulose membranes (1 h at 100 V at 4°C). Membranes were blocked in 7.5% BSA and incubated with the indicated primary antibodies ON followed by appropriate secondary antibodies (LiCor) for 1 h at RT. Image acquisition was performed using a LiCor Scanner and image analysis performed using ImageJ/FIJI (NIH).

### Primary neurons and PFF transduction

Primary hippocampal neurons were derived from P0-P2 wt or *Snca*-GFP pups or from CD1 E16–E18 embryos (Extended Data Fig. 6-2 only). Postnatal hippocampi were dissected in MEM 10 mm HEPES, 1% penicillin/streptomycin, digested with 1 mg/ml Papain for 30 min, and dissociated with a 1-ml tip in MEM 10% FBS, 2 mm GlutaMax, sucrose, 1% penicillin/streptomycin. Cells were centrifuged at 1000 RPM for 4 min, resuspended in MEM, 2% B27, 2 mm GlutaMax, 0.5% penicillin/streptomycin, and plated at the density of 4.5 × 104/cm^2^. Embryonic hippocampi were processed as previously described (Volpicelli-Daley et al., 2014b).

10.1523/ENEURO.0007-20.2020.f1-1 Extended Data Figure 1-1 Full 9027 Western blotting for Figure 1*E*. Download Figure 1-1, TIF file.

**Figure 2:**
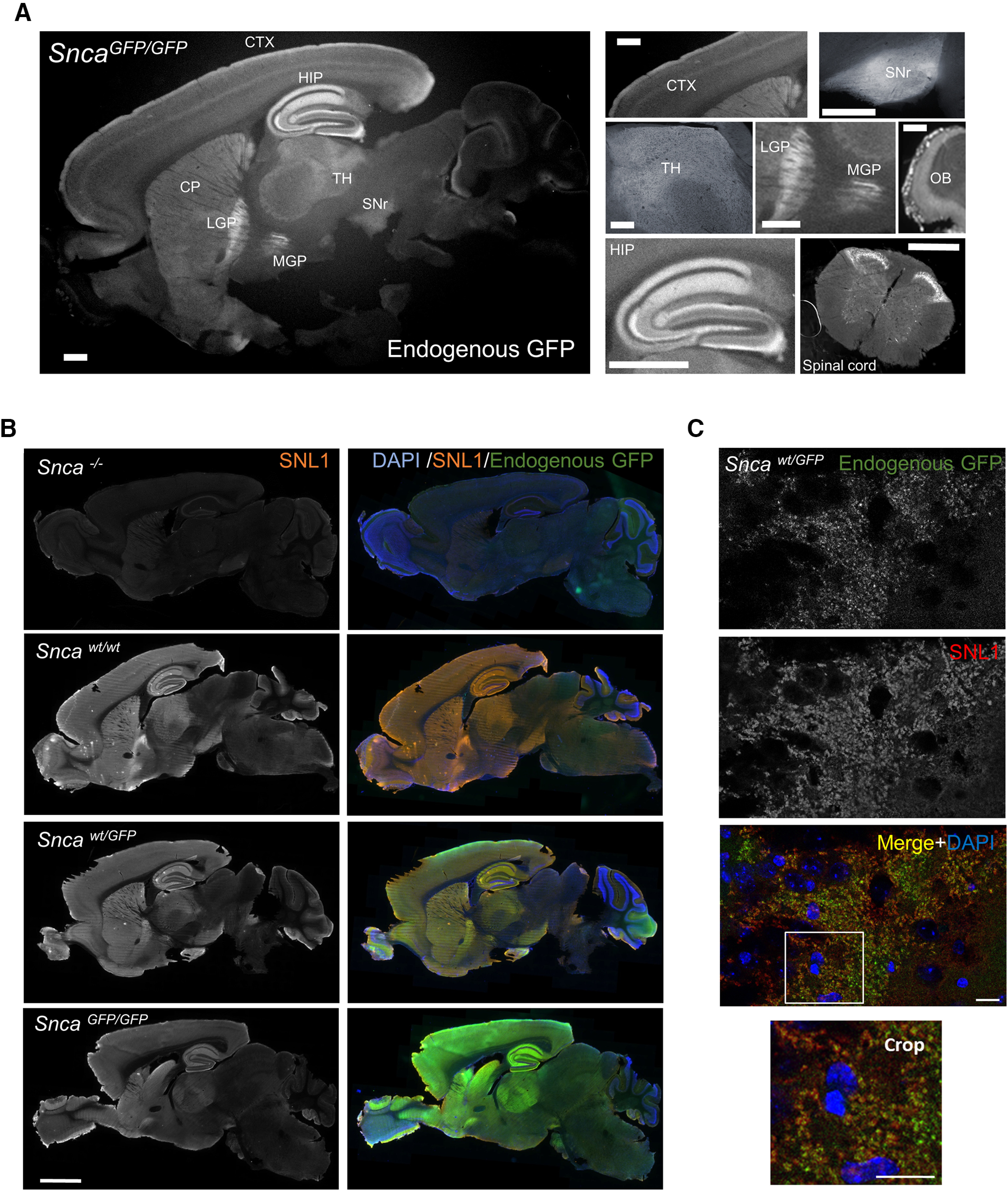
Distribution of aSyn-GFP in brain and spinal cord. ***A***, Expression pattern of aSyn-GFP in the brain from a homozygous (*Snca^GFP/GFP^*) animal. Endogenous GFP fluorescence is shown. Individual brain areas are shown on the right. ***B***, Immunofluorescence showing aSyn expression patterns in wt, *Snca^wt/GFP^*, *Snca^GFP/GFP^*, and *Snca*^−/−^ mice using a pan-aSyn antibody (SNL1). ***C***, Co-localization of aSyn-GFP and endogenous aSyn in the hippocampus (CA3) of a *Snca^wt/GFP^* mouse labeled with SNL1. CTX = cortex; HIP = hippocampus; CP = caudate putamen; LGP = lateral globus pallidus; MGP = medial globus pallidus; TH = thalamus; SNr = substantia nigra pars reticulata; OB = olfactory bulb; SI = substantia innominata. Scale bars: 1 mm (***A***), 2 mm (***B***), 20 µm (***C***).

10.1523/ENEURO.0007-20.2020.f1-2 Extended Data Figure 1-2 Coomassie staining (left) and Western blotting with the anti-aSyn antibody (Syn9027, right) of different amounts of recombinant mouse aSyn and aSyn-GFP. Download Figure 1-2, TIF file.

**Figure 3.**
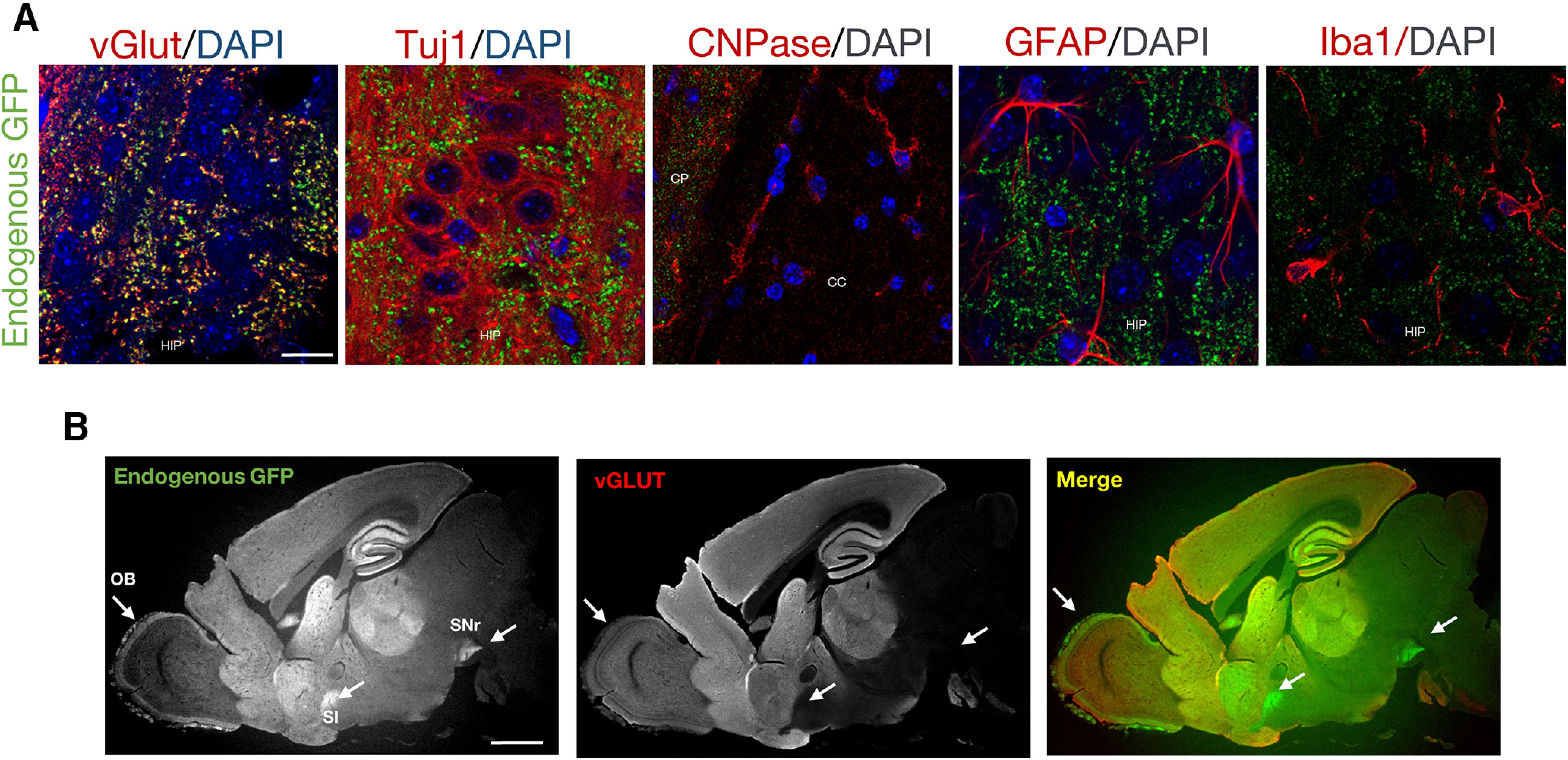
Expression of aSyn-GFP in different CNS cell types. ***A***, Co-localization between aSyn-GFP and markers for neurons (vGlut and Tuj1), oligodendrocytes (CNPase), astrocytes (GFAP), and microglia (IBA1) in a *Snca^GFP/GFP^* mouse. aSyn-GFP is expressed in neurons (Tuj1-positive cells) and enriched in synaptic vesicles containing vGlut. HIP = hippocampus; CP = caudate putamen; CC = corpus callosum. Scale bar: 10 µm. ***B***, Sagittal brain section from a *Snca^GFP/GFP^* mouse stained with an antibody against vGlut. Arrows indicate brain areas in which aSyn-GFP signal is enriched in regions with low vGlut expression. OB = olfactory bulb; SI = substantia innominata; SNr = substantia nigra pars reticulata. Scale bar: 1 mm.

10.1523/ENEURO.0007-20.2020.f1-3 Extended Data Figure 1-3 Western blotting (Syn9027) of brain homogenates of the indicated genotype loaded along with the indicated amount of recombinant aSyn and aSyn-GFP proteins. Download Figure 1-3, TIF file.

PFF transduction was conducted at 10 DIV by adding 2 μg/ml (96-well plate and coverslips) or 10 μg/ml (biochemistry) of PFFs diluted in PBS and sonicated with a BioRuptor (Diagenode) bath sonicator for 10 cycles of 30 s ON, 30 s OFF, high setting. Cells were processed 7 or 14 d post-transduction (DPT).

### Live imaging

*Snca^wt/GFP^* neurons were cultured on MatTek dishes for 10 d, transduced with 2 µg of wt PFFs, and incubated for five additional days. Neurons were then transferred to the imaging chamber (5% CO_2_, 37°C) and allowed to equilibrate for 30 min. Five fields of view were selected and images were taken every 15 min using a Leica DMI 6000 microscope.

### Intracerebral injection of PFFs

A total of 2.5 μl of 2 mg/ml of sonicated mouse wt PFFs were stereotaxically injected in the hippocampus (coordinates: −2.5 mm relative to bregma; 2 mm from midline; 2.4 mm beneath the skull; Fig. 8) of male mice of the indicated genotype (two to four months of age, three mice/genotype; Zhang et al., 2019). Mice were perfused 30 d postsurgery. Additionally, as shown in Extended Data Figures 8-1, 8-2, PFFs were stereotaxically injected into the ventral striatum (AP: +0.2 mm bregma, lateral: 2.0 mm from midline, depth: 3.6 mm beneath the dura), dorsal striatum (AP: +0.2 mm, lateral: 2.0 mm, depth: 2.6 mm), and overlaying cortex (AP: +0.2 mm, lateral: 2.0 mm, depth: 0.8 mm) of mice (females) of the indicated genotype and mice were perfused at 30 (one mouse/genotype; data not shown) or 90 d postinjection (one mouse/genotype).

### RT-PCR

Mice were perfused, brain removed in conditions that minimize RNA degradation, and half hemispheres were frozen for further processing. Frozen tissue was thawed and immediately homogenized in 2 ml of RTL buffer + 2 mm DTT (QIAGEN). mRNA was extracted from 300 μl of homogenate using the RNeasy kit (QIAGEN) according to manufacturer’s instructions; 200–800 μg of mRNA were converted to single strand cDNA with SuperScript III First-Strand Synthesis System (Thermo Fisher Scientific) using random hexamers and the protocol detailed by the vendor. Brain derived cDNA and primers targeting mouse aSyn (Table 1) were used in Syber Green (Thermo Fisher Scientific) real-time PCR reactions monitored by a 7500 Fast Real Time PCR system (Applied Biosystems). Actin and SNAP 25 were used as internal controls (Table 1). Data are expressed as fold change over the wt genotype.

### Measurements of GFP signal in mouse blood

Total blood was collected in EDTA containing tubes by cardiac puncture in deep terminal anesthesia and right before transcardial perfusion (see specific section); 25 μl of blood were lysed by addition of 225 μl of lysis buffer, and 5 μl of lysate were diluted in 190 μl of water and analyzed for fluorescence using a spectrophotometer (excitation: 488 nm; emission: 530 nm).

### Experimental designs and statistical analysis

Details for each experiment and statistical analysis are described in the figure legends. Statistical analysis was performed using GraphPad Prism (v7) or GraphPad Prism (v8 for Fig. 7*C*).

## Results

### *Snca-GFP* knock-in (KI) mice express wt levels of aSyn-GFP fusion protein

We generated a novel mouse line that genetically encodes fluorescent aSyn by using homologous recombination to insert the cDNA for enhanced-GFP into the *Snca* locus of murine embryonic stem cells (Fig. 1*A*; see also Materials and Methods). The construct was targeted to the 3′ end of exon 6 while keeping the surrounding endogenous regulatory elements intact. The modified *Snca-GFP* gene was predicted to transcribe a fusion protein consisting of wt murine aSyn with a C-terminal GFP-tag. Embryonic stem cells containing the recombined construct following electroporation were confirmed by Southern blot analysis (Fig. 1*A*, right panel), and a validated clone (1B6) was implanted in C57BL/6 blastocysts to yield founder lines. Two founder mouse lines were derived, and offspring with normal karyotypes were further expanded by breeding out onto a C57BL/6 background. PCR genotyping with primers targeting exon-intron boundaries revealed mice with either wt, heterozygous (*Snca^wt/GFP^*), or homozygous (*Snca^GFP/GFP^*) KI genotypes (Fig. 1*B*). Both KI genotypes were fertile and did not show detectable differences in lifespan nor any overt behavioral defects up to two years of age.

Upon illumination with blue light, brains and spinal cords from *Snca^wt/GFP^* and *Snca^GFP/GFP^* mice fluoresced brightly, in line with expression of the aSyn-GFP fusion protein in tissues where aSyn is normally abundant (Fig. 1*C*). Quantification of aSyn mRNA by RT-PCR showed that total transcript levels did not differ between KI and wt mice (Fig. 1*D*). We further confirmed that this represented the expression of an intact aSyn-GFP fusion protein by subjecting brain homogenates from wt and KI mice to immunoblot analysis. Probing with antibodies against aSyn and GFP showed a 45-kDa product corresponding to the predicted molecular weight of aSyn-GFP in *Snca^GFP/GFP^* and *Snca^wt/GFP^* littermates that was absent from wt controls (Fig. 1*E*). Furthermore, no significant cleavage products were detected by either antibody. However, a monoclonal antibody to aSyn (Syn9027; Fig. 1*E*; Extended Data Figs. 1-1, 1-2, 1-3) and a rabbit polyclonal antibody (SNL1; data not shown) both showed stronger reactivity for aSyn-GFP relative to the untagged form by immunoblot. Nonetheless, *Snca ^wt/GFP^* mice expressed both aSyn-GFP and untagged aSyn at ∼50% of the amount of aSyn seen in *Snca ^GFP/GFP^* and wt mice, respectively (Fig. 1*E*, lower panel). These results indicate that *Snca-GFP* mice express physiological levels of an aSyn-GFP fusion protein with minimal disruption of endogenous mouse aSyn mRNA and protein levels.

### Regional and subcellular localization of aSyn-GFP in *Snca-GFP* mice is identical to wt animals

Since the endogenous promoter and regulatory elements remain unmodified in *Snca-GFP* mice, we predicted that the regional and subcellular distribution of aSyn-GFP would be comparable between wt and both KI genotypes. Examination of native GFP fluorescence in 40-µm-thick brain and spinal cord sections revealed abundant aSyn-GFP expression particularly in the hippocampus, substantia nigra, striatum, cerebral cortex, globus pallidus, thalamus, and olfactory bulb (Fig. 2*A*), mirroring the endogenous pattern previously reported for C57BL/6 mice (Taguchi et al., 2016). Detection by immunofluorescence also showed no obvious differences in regional aSyn distribution between sections from *Snca^wt/wt^*, *Snca^wt/GFP^*, and *Snca^GFP/GFP^* mice (Fig. 2*B*). In addition, GFP-fluorescence overlapped almost completely with that of aSyn in *Snca^wt/GFP^* mice, further indicating the intact tagged protein is normally expressed and does not perturbate the distribution of wt aSyn (Fig. 2*C*).

Co-immunostaining of GFP with markers for different CNS cell types showed that aSyn-GFP is expressed by neurons (Tuj1-positive) but is not detectable in astrocytes (GFAP-positive), oligodendrocytes (CNPase-positive) or microglial (Iba1-positive) cells (Fig. 3*A*). Within neurons, aSyn-GFP co-localized with glutamate vesicular transporter (vGLUT; Fig. 3*A*,*B*), a presynaptic marker, but not with Tuj1, consistent with the known enrichment of aSyn in presynaptic vesicles in excitatory neurons (Maroteaux et al., 1988; Burre, 2015). aSyn-GFP was also detectable in brain areas containing non-glutamatergic neurons (e.g., striatum and olfactory bulb; Fig. 3*B*), where it co-localized with glutamate decarboxylase (GAD) positive neurons (data not shown). Together, these results suggest that the *in vivo* distribution of the aSyn-GFP fusion protein closely matches that of wt aSyn at the regional, cellular, and subcellular levels.

### aSyn-GFP is enriched in presynaptic vesicles in *Snca-GFP* primary neurons

**Figure 4.**
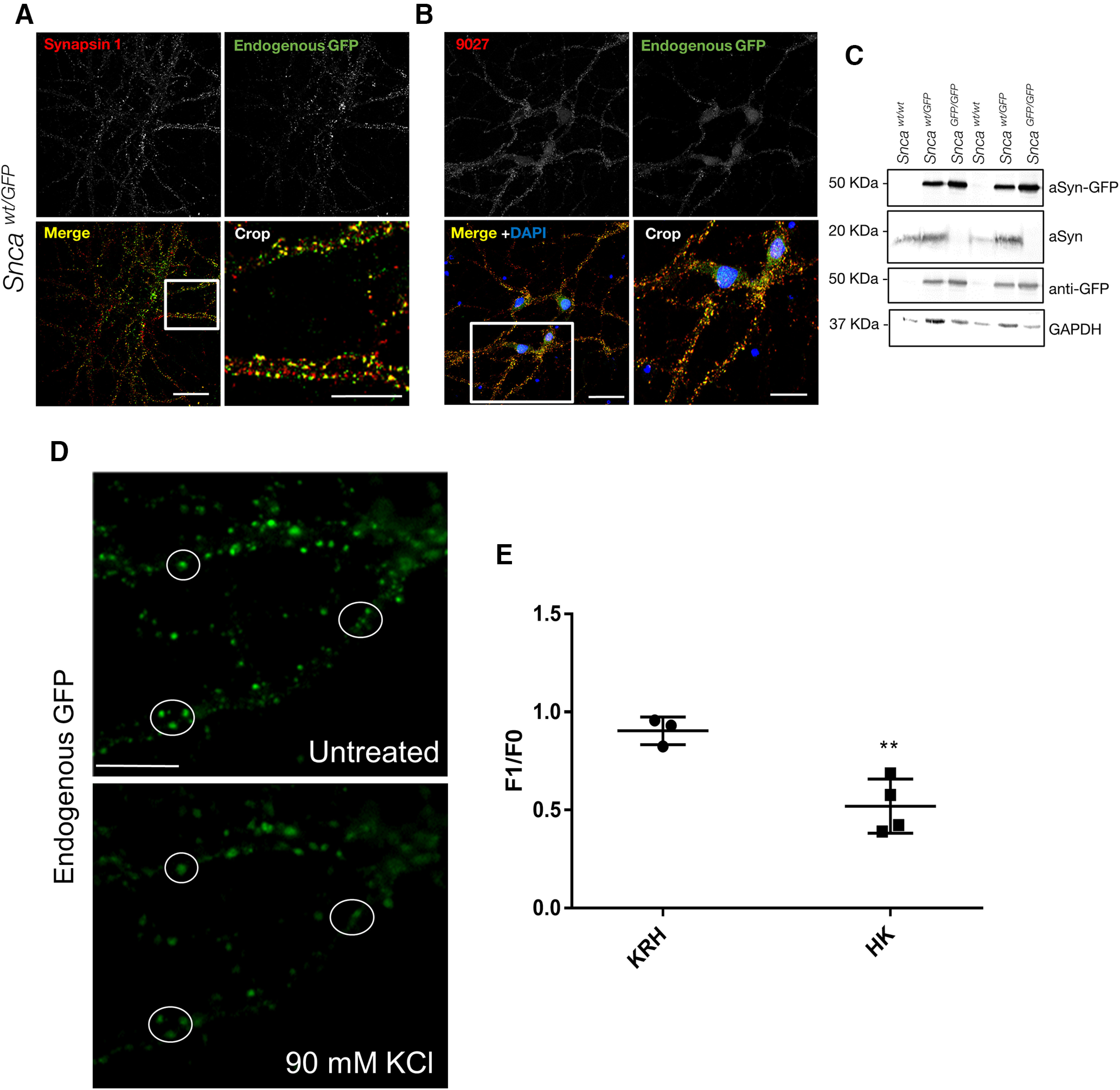
Expression, subcellular localization and synaptic vesicle cycle of aSyn-GFP in primary neurons. Primary hippocampal neurons were derived from newborn pups and kept in culture for 14–21 d. ***A***, Co-localization of aSyn-GFP with the presynaptic marker Synapsin 1. ***B***, Co-localization between endogenous GFP signal and an anti-aSyn antibody (9027) in *Snca^wt/GFP^* neurons. Immunocytochemistry showing distribution of aSyn in wt neurons is shown in Extended Data Figure 4-1. ***C***, Western blotting of lysates from neurons of the indicated genotypes using antibodies against aSyn, GFP, and GAPDH. Complete blot is shown in Extended Data Figure 4-2. ***D***, Heterozygous primary hippocampal neurons were imaged before and after stimulation with 90 mm KCl. ***E***, Quantification of fluorescence intensity of GFP positive vesicles before (F0) and following (F1) treatment with control (KRH, Krebs Ringer HEPES) or stimulation buffer (HK, 90 mm KCl). The data are expressed as the F1/F0 ratio (mean ± SEM from two subfields per dish, >70 vesicles per dish, *N* = 3–4 dishes from two to three independent cultures). Asterisks indicate statistical significance (*p* = 0.0061) as determined by two-tailed unpaired Student’s *t* test with Welch’s correction. Comparison of aSyn and Synapsin 1 redistribution by immunostaining is shown in Extended Data Figure 4-3. Scale bars: 20 µm (***A***, ***B***), 10 µm (crop), 10 µm (***D***).

To further determine the utility of these KI mice for studying aSyn pathobiology, we compared the expression levels and subcellular localization of aSyn-GFP in primary neurons derived from *Snca^wt/GFP^*, *Snca^GPF/GFP^*, and wt mice. Hippocampal and cortical neurons (Fig. 4; data not shown) prepared from postnatal *Snca^wt/GFP^* and *Snca^GPF/GFP^* mice developed normally in culture, with aSyn-GFP detectable by DIV7. aSyn-GFP was mainly enriched in vesicles (Fig. 4*A*), similar to what is seen in *Snca-GFP* brains. As previously reported for wt aSyn, aSyn-GFP was also present in the cell body during early developmental stages (Extended Data Fig. 4-1). Both wt aSyn and aSyn-GFP expression in cultured neurons increased with age before plateauing at DIV10, indicating that the addition of the GFP-tag does not alter the developmentally regulated expression of aSyn in neurons (data not shown). In mature cultures, aSyn-GFP colocalized strongly with Synapsin 1, a marker of synaptic vesicles (Fig. 4*A*). In addition, aSyn and aSyn-GFP strongly overlap in *Snca^wt/GFP^* neurons (Fig. 4*B*). Therefore, neurons prepared from *Snca-GFP* mice exhibit the expected synaptic distribution of aSyn and Synapsin 1.

10.1523/ENEURO.0007-20.2020.f4-1 Extended Data Figure 4-1 Mature *Snca^wt/wt^* primary hippocampal neurons were stained with the same anti-aSyn antibody as in Figure 4 (9027). The staining confirms the cell body and vesicular localization of untagged aSyn. Scale bar: 10 µm. Download Figure 4-1, TIF file.

10.1523/ENEURO.0007-20.2020.f4-2 Extended Data Figure 4-2 Full 9027 Western blotting for Figure 4*C*. Download Figure 4-2, TIF file.

10.1523/ENEURO.0007-20.2020.f4-3 Extended Data Figure 4-3 *Snca^wt/GFP^* neurons were fixed before, after a 2-min exposure to 90 mm KCl, or after a 15-min recovery from HK stimulation and co-stained with an anti-Synapsin 1 antibody. Scale bar: 10 µm. Download Figure 4-3, TIF file.

Immunoblot analysis of hippocampal culture lysates with aSyn and GFP antibodies confirmed the expression of a single major species consistent with intact aSyn-GFP at the expected ratios with respect to the wt protein in each genotype (Fig. 4*C*; Extended Data Fig. 4-2). Taken together, aSyn-GFP maintains normal subcellular expression and distribution without disrupting the morphology of synaptic vesicles.

### aSyn-GFP participates in the synaptic vesicle cycle with kinetics similar to wt aSyn

To further establish that aSyn-GFP has similar functional properties to wt aSyn, we examined its ability to participate in synaptic vesicle cycling in primary hippocampal neurons. In wt neurons, aSyn is dispersed following stimulation with 90 mm KCl and partially repopulates into vesicles after stimulus removal (Fortin et al., 2005). We therefore performed live imaging on DIV18–DIV22 Snca^wt^*^/GFP^ hippocampal neurons to monitor the redistribution of aSyn-GFP during stimulation. Addition of KCl induced a marked decrease in aSyn-GFP intensity in individual vesicles within 2 min of treatment (Fig. 4*D*,*E*), in agreement with the dynamics previously reported for wt aSyn (Fortin et al., 2005). Similar results were observed in *Snca^GFP/GFP^* cultures (data not shown). Neurons were also fixed and immunostained for Synapsin 1, which is known to disperse following stimulation and redistribute back to synaptic vesicles shortly after the stimulus end (Chi et al., 2001). Unlike Synapsin 1, aSyn-GFP did not fully re-establish its vesicular localization after a 15-min recovery period following KCl treatment (Extended Data Fig. 4-3). These results further support that aSyn-GFP is functionally similar to its wt counterpart.

### aSyn-GFP is detectable in multiple peripheral cell types

**Figure 5.**
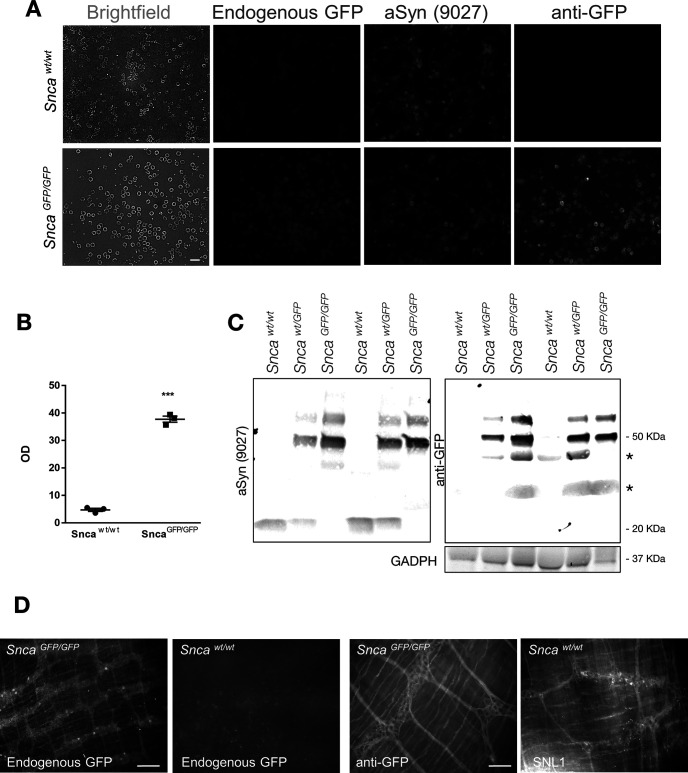
Expression of aSyn-GFP in peripheral cells. ***A***, Total blood from *Snca^wt/wt^* or *Snca^GFP/GFP^* mice visualized using either endogenous GFP fluorescence, an anti-GFP, or anti-aSyn antibody (9027). ***B***, Spectrophotometric reading of GFP signal from lysed total blood from either *Snca^wt/wt^* or *Snca^GFP/GFP^* mice. Data are shown as mean ±SEM, and statistical significance is indicated by asterisks (two-tailed unpaired *t* test with Welch’s correction; *N* = 3 animals/genotype, *p* < 0.0001). ***C***, Western blotting confirmation of the expression of aSyn-GFP in red blood cells. Asterisks denote non-specific bands. ***D***, aSyn-GFP in the myenteric plexus of the colon of unfixed whole mounts from *Snca^GFP/GFP^* mice detected using endogenous fluorescence (left) or an anti-GFP antibody in fixed samples (right). Wt aSyn in *Snca^wt/wt^* mice was detected in fixed samples (right) using an aSyn antibody (SNL-1; *N* = 3 per genotype). Scale bars: 10 µm (***A***), 100 µm (***D***, left), 50 µm (***D***, right).

Since expression of aSyn-GFP in *Snca-GFP* mice remains under the control of the endogenous promoter and regulatory elements, we predicted that its distribution in peripheral organs would also parallel that of the wt protein. We therefore surveyed aSyn-GFP expression in a variety of organs across multiple anatomic regions. As previously reported for wt aSyn (Barbour et al., 2008), GFP fluorescence was prominent in blood (primarily erythrocytes; Fig. 5*A*) and bone marrow (data not shown). Endogenous GFP fluorescence in blood was also detectable in *Snca^GFP/GFP^*mice by spectrophotometry (Fig. 5*B*) and confirmed by immunoblotting in lysates prepared from sedimented red blood cells (Fig. 5*C*). We also detected GFP fluorescence in unfixed colon samples (Fig. 5*D*, left panel) in which aSyn-GFP could be found in neurons of the myenteric plexus. Although the native GFP signal was lost after fixation, expression of the fusion protein was confirmed in this tissue using an anti-GFP antibody (Fig. 5*D*, right panels). In contrast, GFP fluorescence in most other fixed tissues tested was indistinguishable from wt mice suggesting levels below our detection limit, consistent with previous studies.

### GFP-tag partially inhibits but does not abolish aSyn fibril assembly

**Figure 6.**
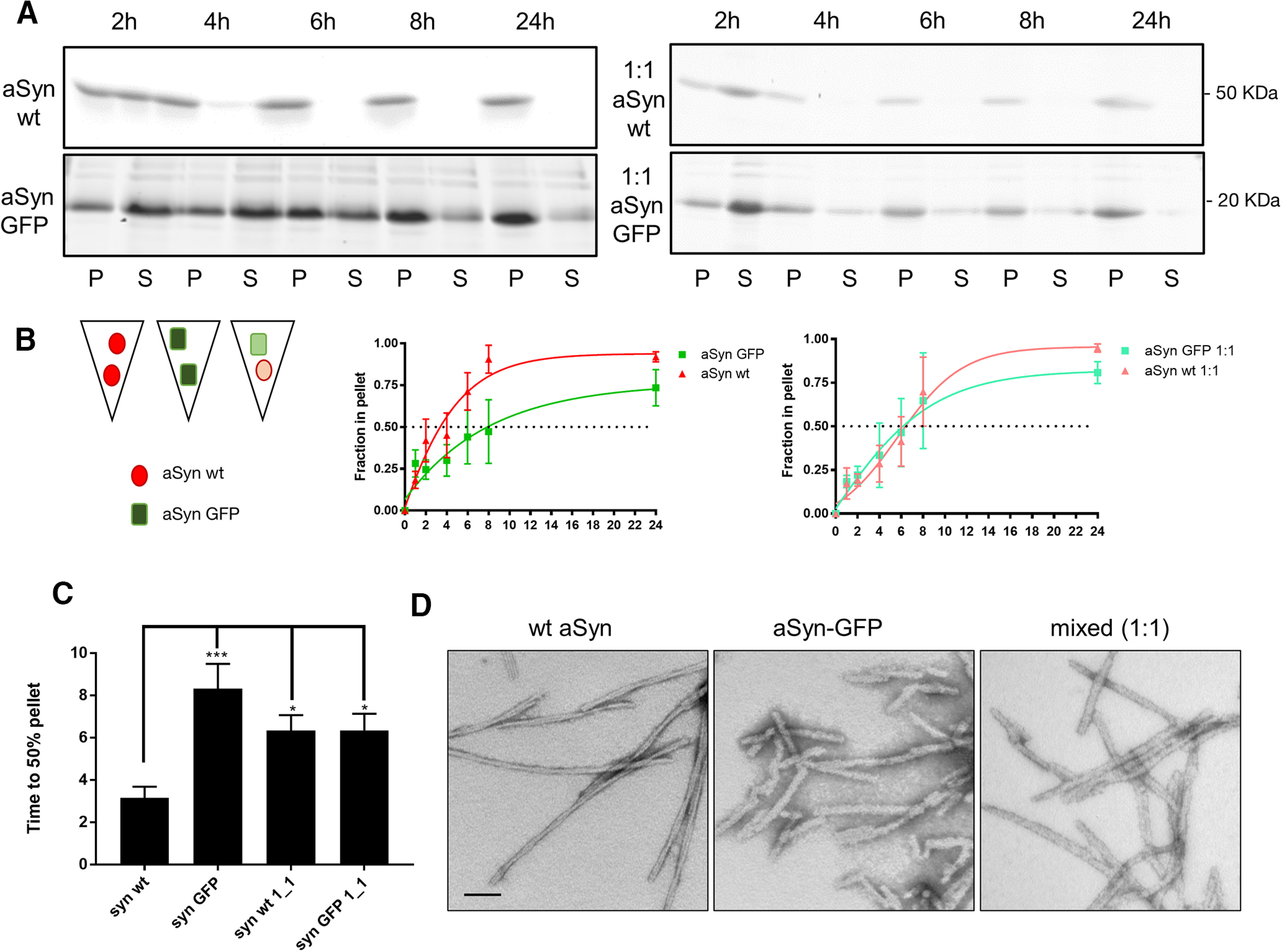
aSyn-GFP is assembly-competent *in vitro*. ***A***, Recombinant wt, aSyn-GFP or a (1:1) mixture of both were agitated for the indicated times at 37°C. Insoluble pellet (P) and soluble supernatant (S) fractions were separated after ultracentrifugation and visualized by Coomassie Blue staining after SDS-PAGE. ***B***, Quantification of data in ***A***, expressed as the relative percentage of protein in the pellet fraction over time. Data (*N* = 3–5 per time point, from two different batches of monomers) were fitted to a sigmoidal curve. Complete blot is shown in Extended Data Figure 6-1. ***C***, Time to 50% pellet (absolute EC_50_) was calculated after fitting the data (mean+ SEM) to a sigmoidal curve. One-way ANOVA with Tukey’s *post hoc* test was used to analyze statistical significance; *p < 0.05, ***p = 0.002. ***D***, Transmission electron microscopy images of negatively stained fibrils from the indicated monomer(s) after 7 d of shaking show fibril formation from all the monomers tested (*N* = 4). Pathogenicity of aSyn-GFP PFFs is shown in Extended Data Figure 6-2. Scale bar: 100 nm.

The normal distribution and functioning of aSyn-GFP in *Snca-GFP* mice led us to examine its utility for investigating the pathobiology of aSyn, specifically its formation into amyloid fibrils that accumulate within intracellular Lewy pathology found in PD and DLB (Arima et al., 1998; Takahashi and Wakabayashi, 2001). Since previous studies showed that the presence of a GFP-tag can alter the rate of aSyn aggregation (Afitska et al., 2017), we tested whether recombinant aSyn-GFP can self-assemble into fibrils under conditions where wt aSyn readily polymerizes. Independent reactions containing either recombinant wt aSyn or aSyn-GFP monomer were incubated with agitation as previously described (Luk et al., 2016). Within 6 h of the start of the reaction, the majority of wt aSyn had converted to insoluble species which accounted for nearly all aSyn by 8 h (Fig. 6*A-C*). In parallel reactions, aSyn-GFP also accumulated in the insoluble fraction over time but with significantly delayed kinetics (Fig. 6*A-C*). When equimolar concentrations of untagged aSyn and aSyn-GFP were combined, both forms aggregated at a similar rate that fell between that of each individual protein (Fig. 6*C*; Extended Data Fig. 6-1). Inspection of the products from these reactions by electron microscopy (Fig. 6*D*), revealed the presence of filamentous structures compatible with those previously reported for aSyn (Luk et al., 2009). Of note, aSyn-GFP fibrils assembled *in vitro* are able to induce aSyn pathology in wt and *Snca*-GFP neurons (Karpowicz et al., 2017; Extended Data Fig. 6-2; data not shown). Taken together, aSyn-GFP is fibril assembly competent, albeit with slower kinetics, which may be mitigated in the presence of untagged aSyn monomer.

10.1523/ENEURO.0007-20.2020.f6-1 Extended Data Figure 6-1 Full Western blottings (Syn9027) for Figure 6*A*, right panels. Download Figure 6-1, TIF file.

10.1523/ENEURO.0007-20.2020.f6-2 Extended Data Figure 6-2 Wt (CD1) primary hippocampal neurons were treated with wt or GFP-tagged aSyn PFFs and stained for phosphorylated aSyn (pSyn) and NeuN 14 d post-PFFs addition. The presence of pSyn signal in aSyn-GFP PFFs-treated neurons demonstrate that these fibrils are competent in inducing aSyn pathology. Scale bar: 20 µm. Download Figure 6-2, TIF file.

### Incorporation of aSyn-GFP into pathologic aggregates following PFF-seeding in *Snca*-GFP neurons

**Figure 7.**
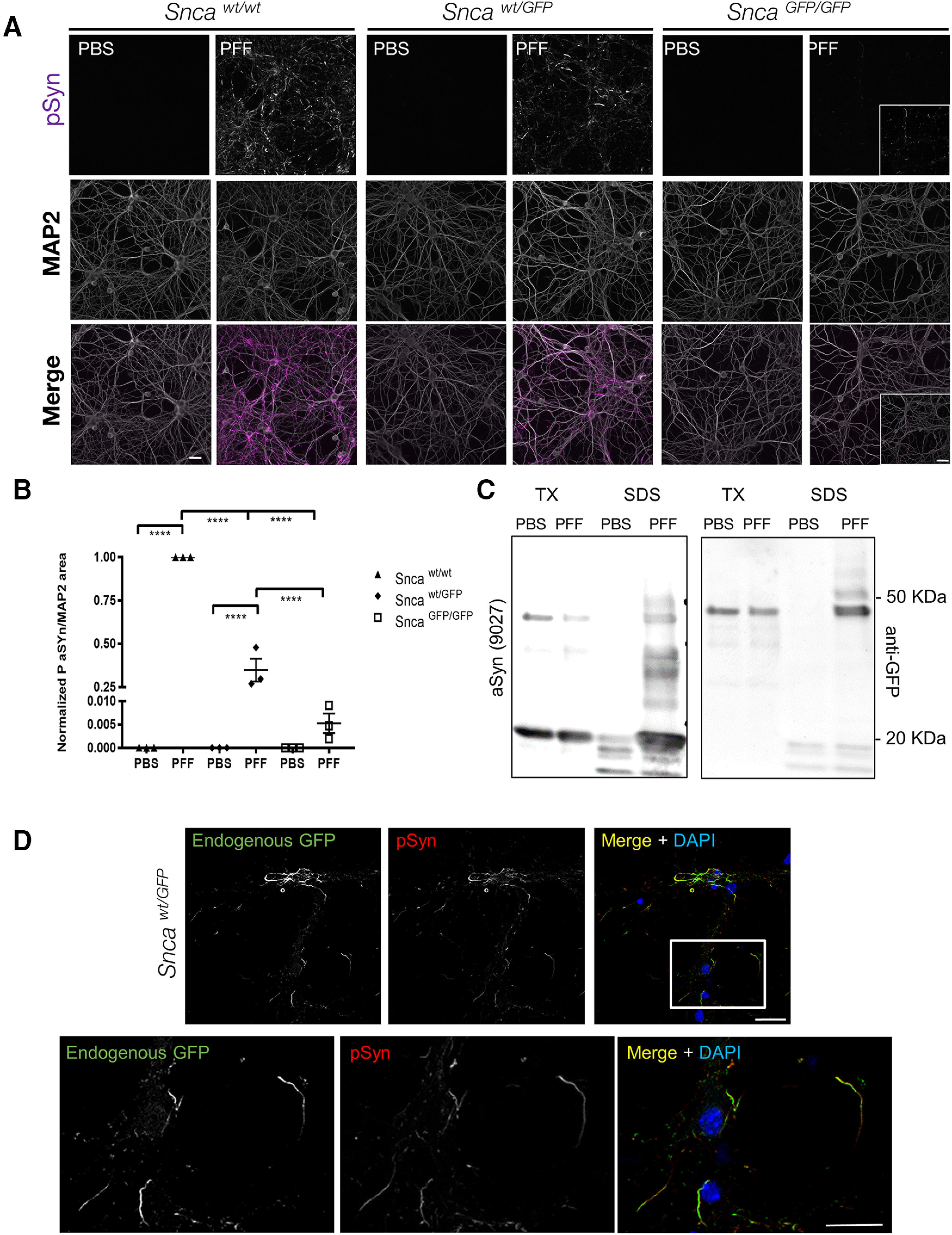
aSyn-GFP forms LB like aggregates in primary neurons. ***A***, Primary hippocampal neurons were treated with mouse wt aSyn PFFs at 10 DIV and phosphorylated synuclein (pSyn) was detected by immunofluorescence after two additional weeks. MAP2 was used to confirm presence of neurons and to normalize pSyn levels. In each experiment, cultures from all three genotypes were present on individual plates and subjected to same treatments and handling. Three to four wells/batch of culture were used and normalized to wt cultures (*N* = 3 from three independent cultures). Live imaging of aSyn-GFP pathology formation is shown in Movie 1. ***B***, Quantification of data in ***A*** normalized to wt cultures (9–12 wells from *N* = 3 independent cultures/genotype; mean ± SEM). Asterisks indicate statistical significance following two-way ANOVA with Sidak’s multiple comparison test; *p* < 0.0001. ***C***, Sequential extraction in 1% TX-100 (TX) and 2% SDS (SDS) of PBS or PFF treated *Snca^wt/GFP^* neurons indicates that aSyn-GFP shifts to the insoluble fraction on PFF exposure. Complete immunoblots are shown in Extended Data Figures 7-1, 7-2. ***D***, Immunostaining of PFF-treated *Snca^wt/GFP^* neurons with an anti-pSyn antibody (81A) following extraction with 1% TX-100, shows nearly complete overlap with endogenous GFP fluorescence (7 d post-PFF addition). Scale bars: 10 µm (***A***), 40 µm (insets), 20 µm (***D***).

**Figure 8.**
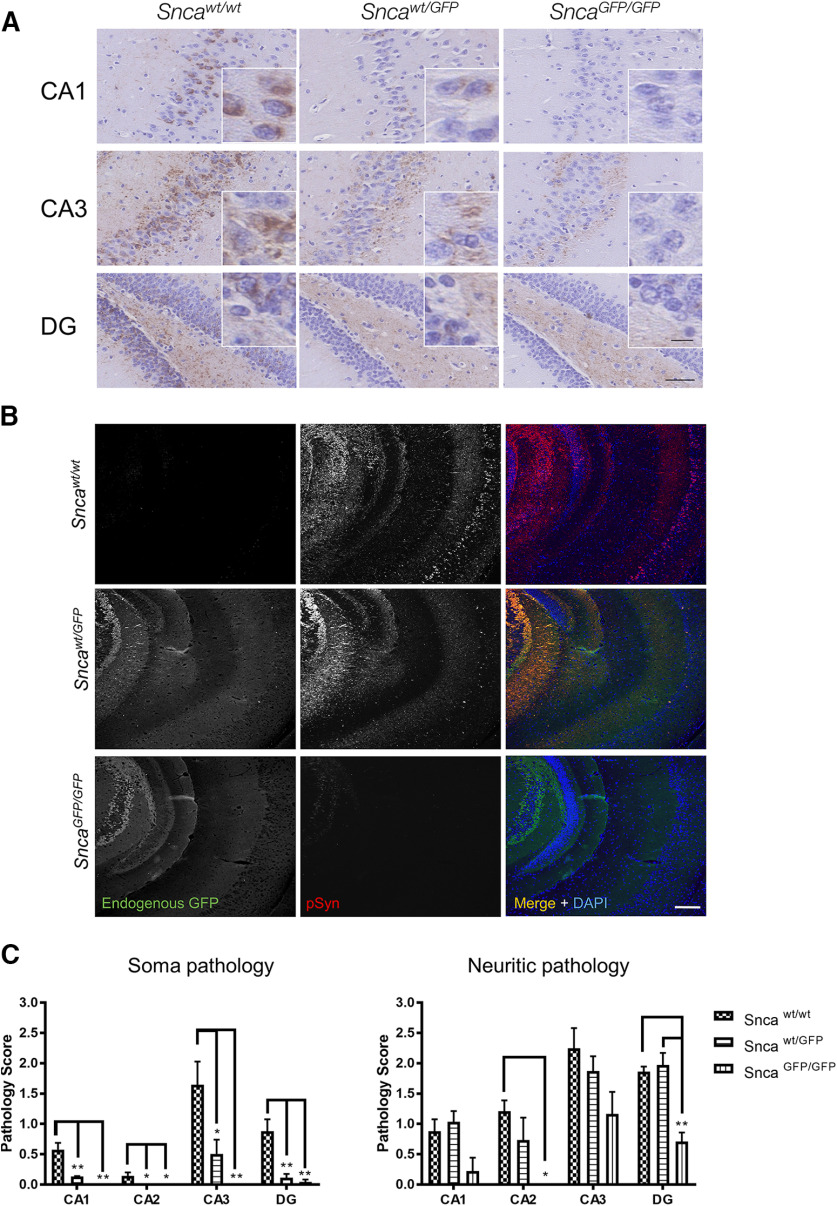
Intracranial injection of aSyn PFFs induces formation of intraneuronal pathology in *Snca^wt/GFP^* mice. Five micrograms of recombinant mouse aSyn PFFs were injected into the hippocampus of *Snca^wt/wt^*, *Snca^wt/GFP^*, or *Snca^GFP/GFP^* mice. Brains were analyzed 30 d postinjection (dpi) by immunohistochemistry using anti-pSyn (81A; ***A***) and immunofluorescence using an anti-pSyn (81A) or anti-GFP antibody (***B***). ***C***, Quantification of the data in ***A***. Plots indicate mean ± SEM. Asterisks indicate statistical significance following one-way ANOVA (brain area) with Tukey’s *post hoc* test; *p < 0.05, **p < 0.005. CA = cornu ammonus; DG = dentate gyrus. Scale bars: 50 µm, 10 µm (crop). Co-localization of PFF-induced pathology in other brain regions are shown in Extended Data Figures 8-1, 8-2, 8-3.

We and others have previously demonstrated that aSyn fibrils internalized by neurons can template the conversion of endogenously expressed aSyn into fibrillar forms that accumulate as Lewy-like inclusions (Volpicelli-Daley et al., 2011). We therefore determined whether primary hippocampal neurons from *Snca-GFP* mice are permissive to such pathologic seeding following exposure to aSyn PFFs and incorporated aSyn-GFP into insoluble intraneuronal inclusions. Neurons from *Snca^wt/GFP^* mice developed inclusions resembling Lewy neurites and Lewy bodies when exposed to mouse wt PFFs, although the level of pathology, as measured by pSer129 aSyn (pSyn) immunostaining, was reduced relative to similarly treated wt neurons (Fig. 7*A*,*B*). In agreement with our *in vitro* data showing that the rate of aggregation is reduced when only aSyn-GFP is present, pathology formation at the same time point was further reduced in *Snca^GFP/GFP^* neurons. Nevertheless, pSyn was still detectable in a small proportion (∼0.005% of wt) of PFF-exposed *Snca^GFP/GFP^*neurons (Fig. 8*A*,*B*).

10.1523/ENEURO.0007-20.2020.f7-1 Extended Data Figure 7-1 Additional Western blotting (Syn9027) of *Snca^wt/GFP^* neurons treated for 14 d with PBS or PFFs and sequentially extracted with 1% TX-100 followed by 2% SDS. Download Figure 7-1, TIF file.

10.1523/ENEURO.0007-20.2020.f7-2 Extended Data Figure 7-2 Western blottings of primary *Snca^wt/GFP^* hippocampal neurons following exposure to PBS or PFFs and sequential extraction with 1% TX-100 (TX) and 2% SDS. Phosphorylation of aSyn-GFP insoluble aggregates was revealed using the MJFR13 antibody. Total aSyn was detected using the anti-aSyn antibody 9027. Download Figure 7-2, TIF file.

In both *Snca^wt/GFP^* and *Snca^GFP/GFP^* neurons, co-labeling with antibodies to pSyn and GFP revealed near-complete co-localization within inclusions following TX-100 extraction (Fig. 7*D*; data not shown), indicating that aSyn-GFP was co-incorporated in phosphorylated aggregates and that aggregates are insoluble. Live imaging of PFF induced GFP containing aggregates in *Snca^wt/GFP^* neurons also show how aSyn-GFP acquires a serpentine like structure and display atypical (compare to aSyn-labeled synaptic vesicles) intracellular motility (Movie 1). Biochemical analysis of TX-100 insoluble proteins from these cultures also confirmed that aSyn-GFP shifted to this fraction after PFF treatment (Fig. 7*C*; Extended Data Fig 7-1) and that the GFP-tagged insoluble material is phosphorylated (Extended Data Fig. 7-2).

Movie 1. **Live imaging of PFF transduced *Snca^wt/GFP^* neurons.** *Snca^wt/GFP^* neurons plated on MatTek^TM^ were transduced at 10 DIV with 2 μg/ml of mouse wild-type PFF and image, 5 days post-transduction, for the indicated time every 15 min at 37°C, in the presence of 5% CO_2_. Arrows indicate vesicular aSyn-GFP and arrowheads serpentine-like or aggregated aSyn -GFP. Scale bar: 10 µm. 10.1523/ENEURO.0007-20.2020.video.1

We further determined whether aSyn-GFP can also undergo pathologic conversion *in vivo* by using intracranial injection to target PFFs into the mouse brain, a model that allows the induction and propagation of aSyn pathology in presence of wt levels of this protein. For this, we selected the hippocampus, because of previous reports (Luna et al., 2018; Nouraei et al., 2018) and our present data showing high levels of aSyn/aSyn-GFP expression in this region. In agreement with this, injection of wt mouse aSyn PFFs into the hippocampus induced the formation of pSyn-positive inclusions within 30 d postinjection (Fig. 8*A*,*B*). Pathology levels were highest in wt mice, followed by *Snca^wt/GFP^*animals, with very low pathology observed in *Snca^GFP/GFP^* mice (Fig. 8*A-C*). Similar results were obtained by injecting PFFs simultaneously into the dorsal striatum, ventral striatum, and overlaying cortex (Extended Data Figs. 8-1, 8-2). In both injection paradigms, pSyn highly co-localized with GFP visualized using an antibody or by its endogenous GFP fluorescence (Fig. 8*B*; Extended Data Fig. 8-2, 8-3), indicating that the fusion protein is incorporated in aSyn aggregates similarly to what we observed in cultured neurons (Fig. 7*A*,*D*). Of note and consistent with the injection data, *Snca*-GFP mice do not develop pathology during normal aging (data not shown) marking a notable difference with previous models overexpressing GFP-tagged aSyn (Hansen et al., 2013).

10.1523/ENEURO.0007-20.2020.f8-1 Extended Data Figure 8-1 Five micrograms of recombinant PFFs were injected into the dorsal striatum, ventral striatum, and cortex of wt, heterozygous, and homozygous *Snca-GFP* mice. Pathology was analyzed at 90 d postinjection (dpi) by immunohistochemistry using an antibody (81A) against pSyn. Scale bars: 50 µm. Download Figure 8-1, TIF file.

10.1523/ENEURO.0007-20.2020.f8-2 Extended Data Figure 8-2 Immunofluorescence using an antibody against pSyn (81A) and an anti-GFP antibody on sections of *Snca^wt/GFP^* mice injected with PFFs in the dorsal striatum, ventral striatum, and cortex show high levels of colocalization in both the substantia nigra and amygdala 90 d postinjection. Scale bars: 100 µm, 50 µm (crop). Download Figure 8-2, TIF file.

10.1523/ENEURO.0007-20.2020.f8-3 Extended Data Figure 8-3 Compresstome brain sections derived from *Snca^wt/GFP^* mice injected in the hippocampus with PFFs (30 d postinjection) and stained with anti-pSyn antibody (81A) show that GFP-tagged aggregates can also be detected using the endogenous fluorescence of the fluorophore. Scale bar: 50 µm. Download Figure 8-3, TIF file.

## Discussion

Clinical and experimental evidence implicate aSyn in multiple neurodegenerative disorders. However, the normal function and regulation of aSyn, along with its role in disease initiation and progression, are not fully understood. Given the dynamic nature of these biological processes, tools that enable the direct visualization of aSyn would facilitate efforts to address these fundamental questions. Moreover, the majority of individuals with synucleinopathy carry neither aSyn mutations nor overexpress aSyn to any measurable extent. To this end, we generated an aSyn-GFP KI mouse line in which endogenous aSyn protein is fused to GFP via its C terminus with the goal of combining the utility of a genetic-encoded fluorescent tag while preserving the protein’s natural distribution throughout the body.

Our data here demonstrate that both heterozygous (*Snca^wt/GFP^*) and homozygous (*Snca^GFP/GFP^*) mice express aSyn-GFP in a pattern that is indistinguishable from aSyn protein in wt animals across multiple tissues including, but not limited to, the central and enteric nervous systems, erythrocytes, and bone marrow. Within the brain, where aSyn is highly enriched, total aSyn mRNA and protein levels were comparable between wt and KI animals, while expression levels of aSyn-GFP were directly proportional to gene dosage (Fig. 1). In contrast to some previous reports, aSyn-GFP appears to be expressed primarily in its intact form and minimal levels of degraded products were detected (McLean et al., 2001). We speculate that expression of aSyn-GFP at endogenous levels and the inclusion of the short linker between aSyn and GFP may contribute to this stability. When examined *in vivo* or in primary neuronal cultures derived from KI mice, aSyn-GFP is detectable only in neurons and is correctly localized to a subset of synaptic vesicles. The almost complete co-localization between native and GFP-tagged aSyn in *Snca^wt/GFP^* mice suggests that aSyn-GFP does not perturb the expression or localization of its wt counterpart (Figs. 2-4). Indeed, the addition of the GFP-tag appears to have a minimal effect on a complex aspect of aSyn function (i.e., participation in synaptic vesicle cycling) as tagged aSyn disperses after synaptic stimulation (Fig. 4), in agreement with previous studies (Fortin et al., 2005; Unni et al., 2010).

The above characteristics distinguish this KI line from previously reported mammalian models that employ a genetically encoded GFP-tag to label aSyn. In these, the tagged sequence corresponds to wt human aSyn with expression regulated by either a non-*Snca* promoter (e.g., PDGFβ; Rockenstein et al., 2005) or downstream of the mouse *Snca* promoter by means of a bacterial artificial chromosome (Hansen et al., 2013). Although robust neuronal expression was achieved in both examples, the distribution and levels of tagged aSyn did not precisely match that of aSyn in the non-transgenic host, and aSyn was also ectopically expressed in additional cell types and regions. Interestingly, GFP-tagged aSyn in these mouse lines also accumulate as lysosome-associated inclusions or undergo phosphorylation at Ser129, a marker of Lewy body and Lewy neurite pathology in human synucleinopathies, with aging. A possible explanation for such differences is that the previously described lines were selected based on high expression levels of the transgene, among other criteria. Total aSyn levels in these mice may have been further amplified by maintaining these mice on a wt genetic background without deletion of the endogenous *Snca* locus. In contrast, the localization and levels of aSyn-GFP in *Snca^wt/GFP^* and *Snca^GFP/GFP^* mice matched that of wt mice, and we did not observe any redistribution or modification of aSyn-GFP in mice up to two years of age. We believe these features make aSyn-GFP KI mice particularly amendable for investigating aSyn trafficking and function, a crucial but understudied area of aSyn biology, especially given that these processes are highly sensitive to aSyn levels (Scott and Roy, 2012; Eguchi et al., 2017).

An added advantage is that aSyn-GFP is normally distributed throughout peripheral organs such as neurons in the gastrointestinal tract and red blood cells in this line (Fig. 5). As demonstrated here, this expression also permits rapid quantification of aSyn levels without the need for additional sample processing (e.g., immunostaining). It is anticipated that this will further enable the validation of endogenous aSyn expression in other peripheral tissues, especially those where aSyn is in low abundance, and where detection using immunohistochemistry alone has provided equivocal results. Primary cells derived from aSyn-GFP KI mice are also a resource for investigating the aSyn biology at the cellular level.

In addition to physiological functioning, our work here demonstrates that *Snca^wt/GFP^* and, to a much lower extent *Snca^GFP/GFP^*, neurons serve as a permissive cellular host for pathologic seeding by misfolded aSyn species. Specifically, recombinant aSyn PFFs induced GFP-positive Lewy-like pathology in cultured hippocampal neurons when introduced into the culture media (Fig. 7) or in multiple CNS regions when PFFs are stereotaxically injected into either dorsal striatum, ventral striatum, and cortex or hippocampus (Fig. 8). Importantly, fibril-induced pathology in *Snca^wt/GFP^* and *Snca^GFP/GFP^* was distributed in the same brain regions as in wt mice, albeit with different densities, suggesting a similar spreading process within neuroanatomical pathways. The intact aSyn-GFP moiety was incorporated in these intraneuronal inclusions (Figs. 7, 8), confirming that this pathology is formed predominantly by aSyn-GFP derived from the neuronal pool and is consistent with previous studies showing that neurons overexpressing aSyn-GFP support fibril-induced pathology formation (Volpicelli-Daley et al., 2014a; Osterberg et al., 2015). We also find no evidence of the existence of intracellular inclusions containing exclusively untagged or tagged aSyn in PFF-treated neurons, although additional studies are required to ascertain the precise proportion of wt and GFP-tagged aSyn within inclusions.

Although our data clearly show that aSyn-GFP can polymerize into fibrils and can be recruited into Lewy-like pathology in neurons, the kinetics of this process is altered by the presence of the GFP-tag. The attenuated levels of pathology induced by PFF treatment, particularly in *Snca^GFP/GFP^* neurons and mice (Figs. 6-8), represent a limitation of using this construct. These observations are in agreement with previous reports and underline the importance of the C terminus domain and protein flexibility in facilitating conversion to a pathologic conformation (McLean et al., 2001; Bertoncini et al., 2005; Afitska et al., 2017), although the constraints brought on by the sterically larger GFP moiety does not abrogate seeding activity. Indeed, aSyn-GFP monomers readily assemble under the same *in vitro* conditions as untagged aSyn and can be incorporated into fibrils at near stoichiometric levels in presence of an equimolar concentration of wt aSyn monomer, with aggregation kinetics that are intermediate relative to wt aSyn or aSyn-GFP alone (Fig. 6). In line with these *in vitro* observations, fibril-induced pathologic seeding in neurons and *in vivo* also show a similar pattern with pathology formation in neurons from wt mice forming inclusions the most rapidly and abundantly, followed by *Snca^wt/GFP^* and then *Snca^GFP/GFP^* mice. Importantly, like in the case of wt aSyn, GFP-containing aggregates appeared to be morphologically distinct from physiological aSyn and hyper-phosphorylated (Figs. 7, 8). Data from *Snca^wt/GFP^* neurons also indicate that recruited aSyn-GFP is detergent insoluble (Fig. 7) and display altered morphology and trafficking (Movie 1). These results support the idea that aggregates containing aSyn-GFP, especially when formed in presence of both tagged and untagged protein, closely resemble wt aSyn pathologic species.

In summary, we have generated a novel *in vivo* resource for studying multiple aspects of aSyn function under physiological and disease-like conditions without genetic overexpression. In addition, *Snca*-GFP mice provide the opportunity to concomitantly track and measure soluble and pathologic forms of the protein across relevant tissues. We therefore anticipate that future studies leveraging these animals (e.g., by crossbreeding with other genetic models of disease) should provide additional insights into aSyn biology.

## Synthesis

Reviewing Editor: Leonidas Stefanis, Biomedical Research Foundation of the Academy of Athens Decisions are customarily a result of the Reviewing Editor and the peer reviewers coming together and discussing their recommendations until a consensus is reached. When revisions are invited, a fact-based synthesis statement explaining their decision and outlining what is needed to prepare a revision will be listed below. The following reviewer(s) agreed to reveal their identity: Poul Jensen, Maria Xilouri. Note: If this manuscript was transferred from JNeurosci and a decision was made to accept the manuscript without peer review, a brief statement to this effect will instead be what is listed below.

The manuscript presents a very interesting new model through which asyn at near endogenous levels can be monitored in real time due to a GFP tag. This model may be very useful to the scientific community. However, some issues, delineated below, need to be addressed in order to improve the presentation of the model and its applicability, especially in the context of seeding.

Fig 1. Whether indeed the AS-GFP protein is present at higher levels than WT AS needs to be assessed properly with both wt-AS and AS-GFP on the same membrane and using more epitope specific AS antibodies. This is particularly important, because, if indeed AS_GFP is present at higher levels than the WT, it suggests, given the similar mRNA levels, that the AS-GFP protein may be handled differently from WT AS by the cells and may have a longer half-life.

Figure 2: Figure legend is not very informative. Scale bars should be indicated in the legend. The particular region of the hippocampus from which the images in C were derived should be indicated. There is no indication regarding the area from which the cropped image arose and no indication regarding the scale bar of the cropped image. In general, all the grayscale presented immunofluorescence images cannot be easily appreciated. In all cases where a double immunostaining is presented (like 2C) it is best to have every antibody with a different color, not grayscale. The colocalization does not appear complete, and could be quantified.

The figure legend in Fig 3 is incomplete. Which brain area is shown in 3A and which genotype was used (Snca wt/GFP or Snca GFP/GFP)? What do the authors show in 3B? What do the arrows indicate? Where is the colocalization with GAD that the authors describe in the results section? The authors should specify that there is no apparent colocalization of GFP with Tuj1, presumably because Tuj1 does not stain the presynaptic terminals, where AS mainly resides. High magnification insert should be provided from the GFP-bright areas of SNr, striatuma and olfactory bulb in panel B, that not is described in the legend.

Fig. 4 panel B demonstrates significant AS and GFP in the cell body that in intensity not differs very much from the presynaptic signals. This needs to be clarified by more antibodies and images and should be compared to staining of wt neurons by the same antibodies. It is important to determine if the anterograde axonal transport of AS-GFP is as efficient as for wt-AS because it will inform about the cellular metabolism of the fusion protein.

In Figure 5, Snca wt/wt animals should be also used to ensure the proper comparison with the GFP-tagged proteins. Costaining of endogenous wt aSyn with synapsin 1 both in Snca wt/GFP and in Snca wt/wt animals should be presented.

Figure 6. Quality of the blots, and in particular the presence of non-specific bands, could be improved.

Fig.7 addresses whether purified recombinant AS-GPF can aggregate like AS. The aSyn-GFP immoblot (bottom panel) in A has 11 samples instead of 10. The data provided demonstrates GFP-AS turns insoluble slower than AS and forms more granular albeit elongated structures in contrast to the slender AS fibrils. Experiments are also conducted where AS and AS-GFP are mixed and subjected to sedimentation assays. The Coomassie blue stained gels must not be cut as in Fig. 7A but presented so that both the AS band at 20 kD and the 50 kD AS-GFP bands are visible on the same gel. Moreover, there need to be conducted both thioflavine and K114 fluorescence spectroscopy of the aggregates to ascertain the AS-GFP aggregates indeed are of an amyloid nature.

In Figure 8A, double immunostaining with paSyn and MAP2 should show clearly the localization of paSyn within MAP-positive processes. It is especially crucial to demonstrates this in the asyn-GFP/a-Syn-GFP neurons, where the p-aSyn staining is very faint, and issues are raised about its specificity. The authors should also demonstrate the GFP fluorescence for those cultures expressing the fusion protein and the aggregates should also be detected using an aggregate specific antibody like the Abcam MJF14.

In Figure 8C, there is only one sample demonstrated, but the assay using protein fractionation will carry some variation. Hence samples in dupli- or triplicates should be analyzed. How big is the reduction in wtAS and AS-GFP in the TX100 soluble fraction upon addition of PFF? The authors should ensure that the results with the GFP antibody do not represent remnants of bands recognized with the aSyn Ab following stripping. A pS129 immunoblot could determine if the SDS-soluble AS-GFP is phosphorylated on Ser129 and to the same extent as the native cellular AS. This information is crucial and needs to be provided. Panel D focuses on colocalisation of pS129 and GFP fluorescence and does not show a perfect match. More high and lower magnification images need to be provided, and there should also be staining by aggregate specific antibodies like e.g. Abcam MJF14 to really convince the readers that AS-GFP can incorporate in or trace aggregates formed in cellulo. In 8D, a square in the merged image must indicate the area from where the cropped image was taken. Cropped images should contain both individual immunotainings and the merged images. Also, scale bars are missing in all panels.

Fig. 9 demonstrates pS129 pathology after in vivo PFF injection into the hippocampus. Wt/wt mice demonstrate the strongest pathology in the nerve cell soma with nearly absent staining in the GFP/GFP mice. However, in the neurites similar pathology occurs in wt/AS-GFP mice, and in CA3 and DG significant pathology occur even in the AS-GFP/AS-GFP. Immunoblots should be provided that demonstrate that the pS129 signal indeed is on the AS-GFP moiety as suggested by the AS-GFP/AS-GFP mouse staining. If this is the case, then it will strengthen the conclusion that even the AS-GFP/AS-GFP model will be useful for studies of templated AS aggregate spreading. In B, fluorescence staining images should also be provided for the conditions with and without PFFs for all 3 groups of mice. For the Extended Figure 9B, a co-staining with a neuronal marker is required. For the nigra, co-staining with TH is preferred.

The authors should be clear about the limitations of the model, especially regarding the limited seeding of GFP-aSyn.
