# Supplementary figures and images for: Snca-GFP Knock-In Mice Reflect Patterns of Endogenous Expression and Pathological Seeding
Source: eNeuro. 2020 Aug 27;7(4):ENEURO.0007-20.2020. doi: 10.1523/ENEURO.0007-20.2020 (PMC7470929; doi:10.1523/ENEURO.0007-20.2020)

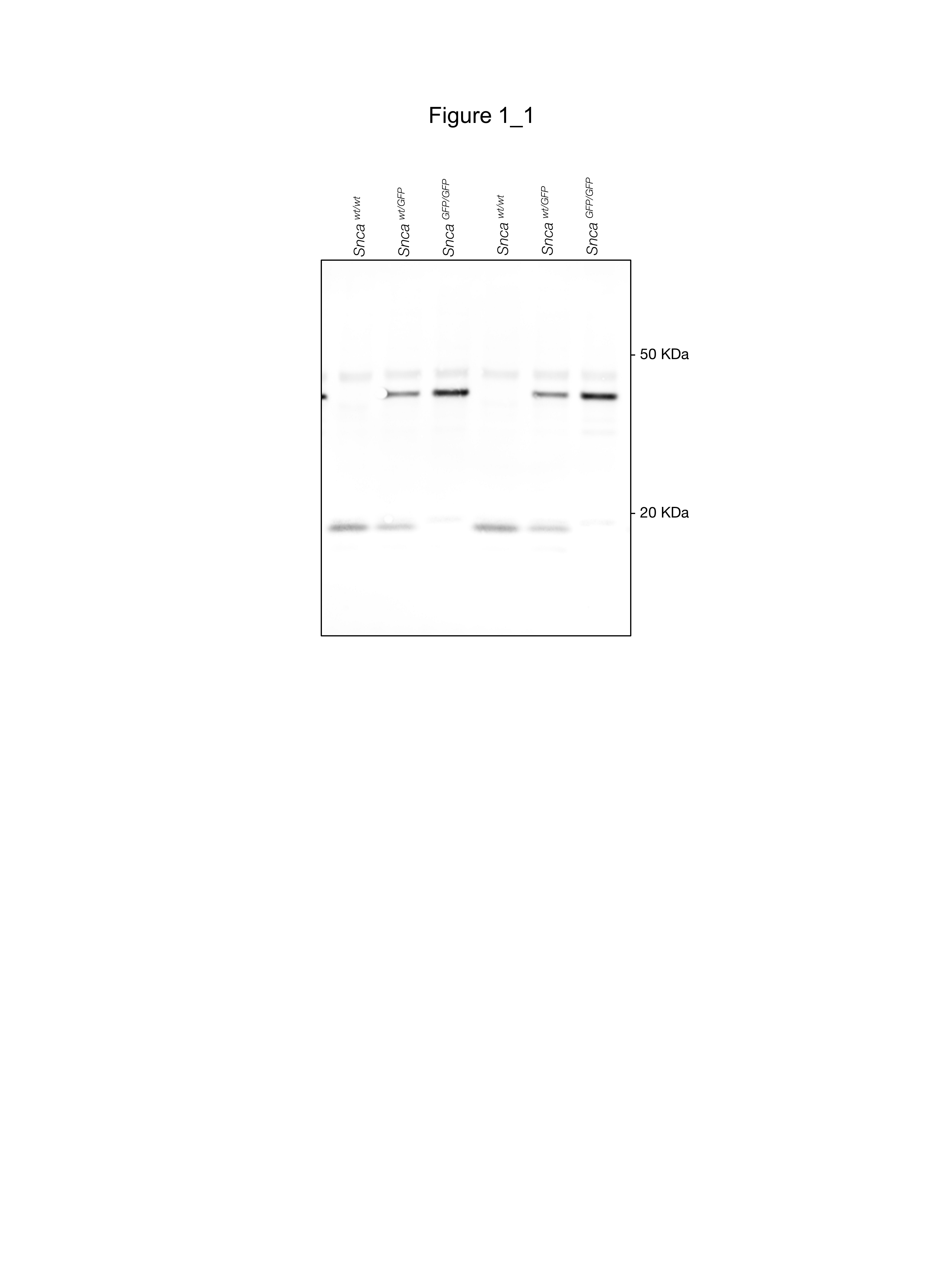

Supplement: Extended Data Figure 1-1 — Full 9027 Western blotting for Figure 1E. Download Figure 1-1, TIF file. [file enu-eN-MNT-0007-20-s01.tif]

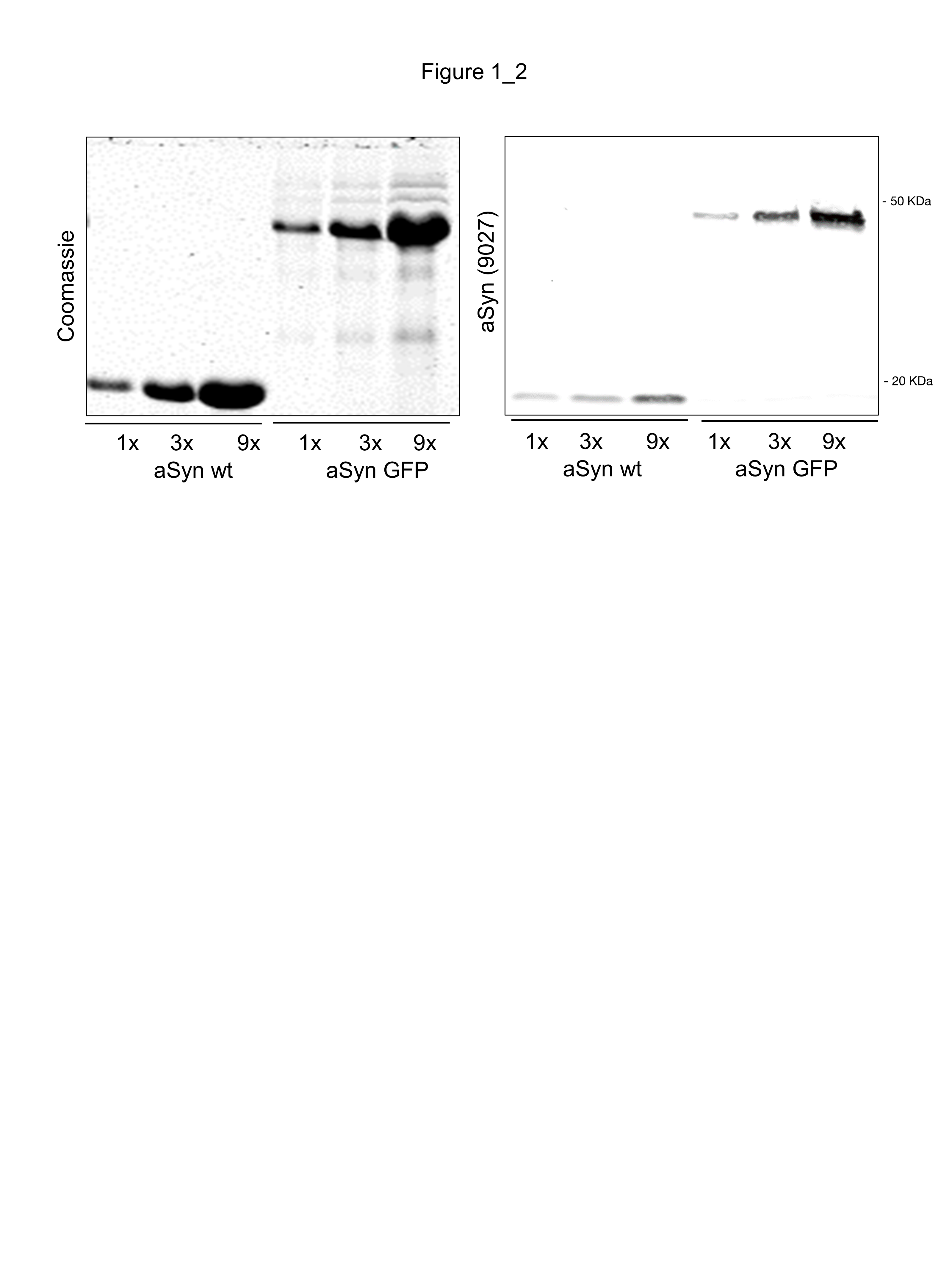

Supplement: Extended Data Figure 1-2 — Coomassie staining (left) and Western blotting with the anti-aSyn antibody (Syn9027, right) of different amounts of recombinant mouse aSyn and aSyn-GFP. Download Figure 1-2, TIF file. [file enu-eN-MNT-0007-20-s02.tif]

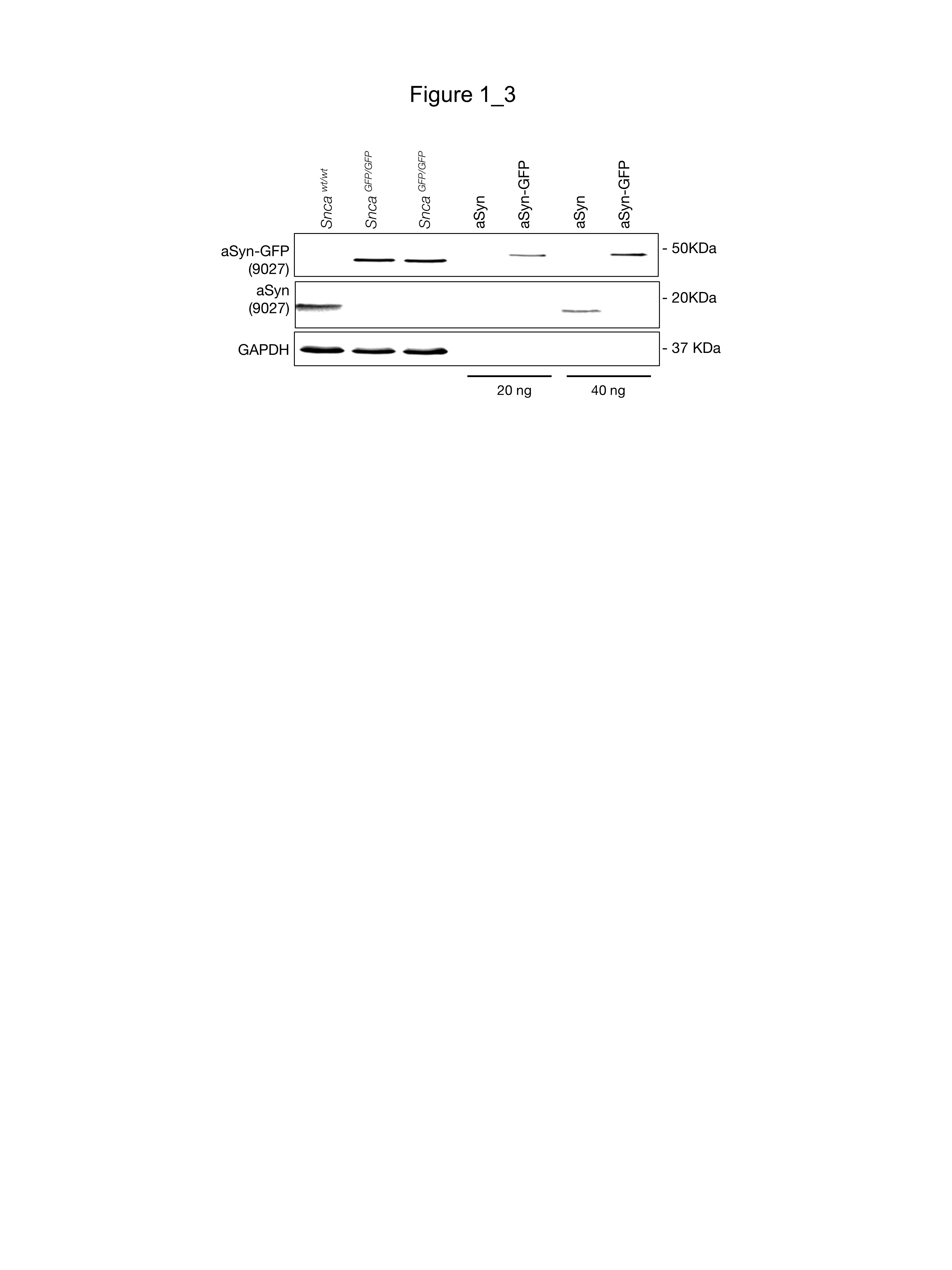

Supplement: Extended Data Figure 1-3 — Western blotting (Syn9027) of brain homogenates of the indicated genotype loaded along with the indicated amount of recombinant aSyn and aSyn-GFP proteins. Download Figure 1-3, TIF file. [file enu-eN-MNT-0007-20-s03.tif]

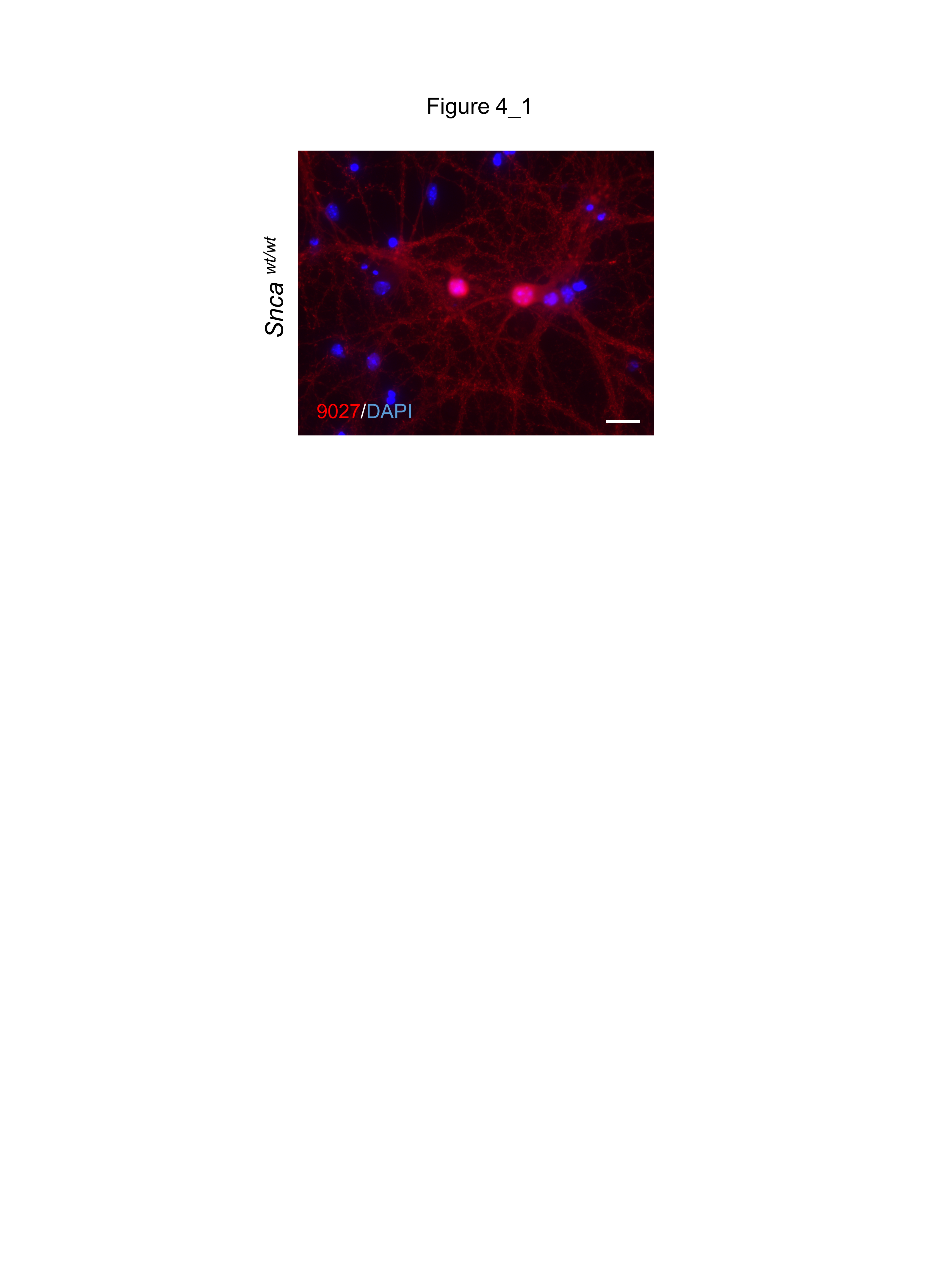

Supplement: Extended Data Figure 4-1 — Mature Sncawt/wt primary hippocampal neurons were stained with the same anti-aSyn antibody as in Figure 4 (9027). The staining confirms the cell body and vesicular localization of untagged aSyn. Scale bar: 10 µm. Download Figure 4-1, TIF file. [file enu-eN-MNT-0007-20-s04.tif]

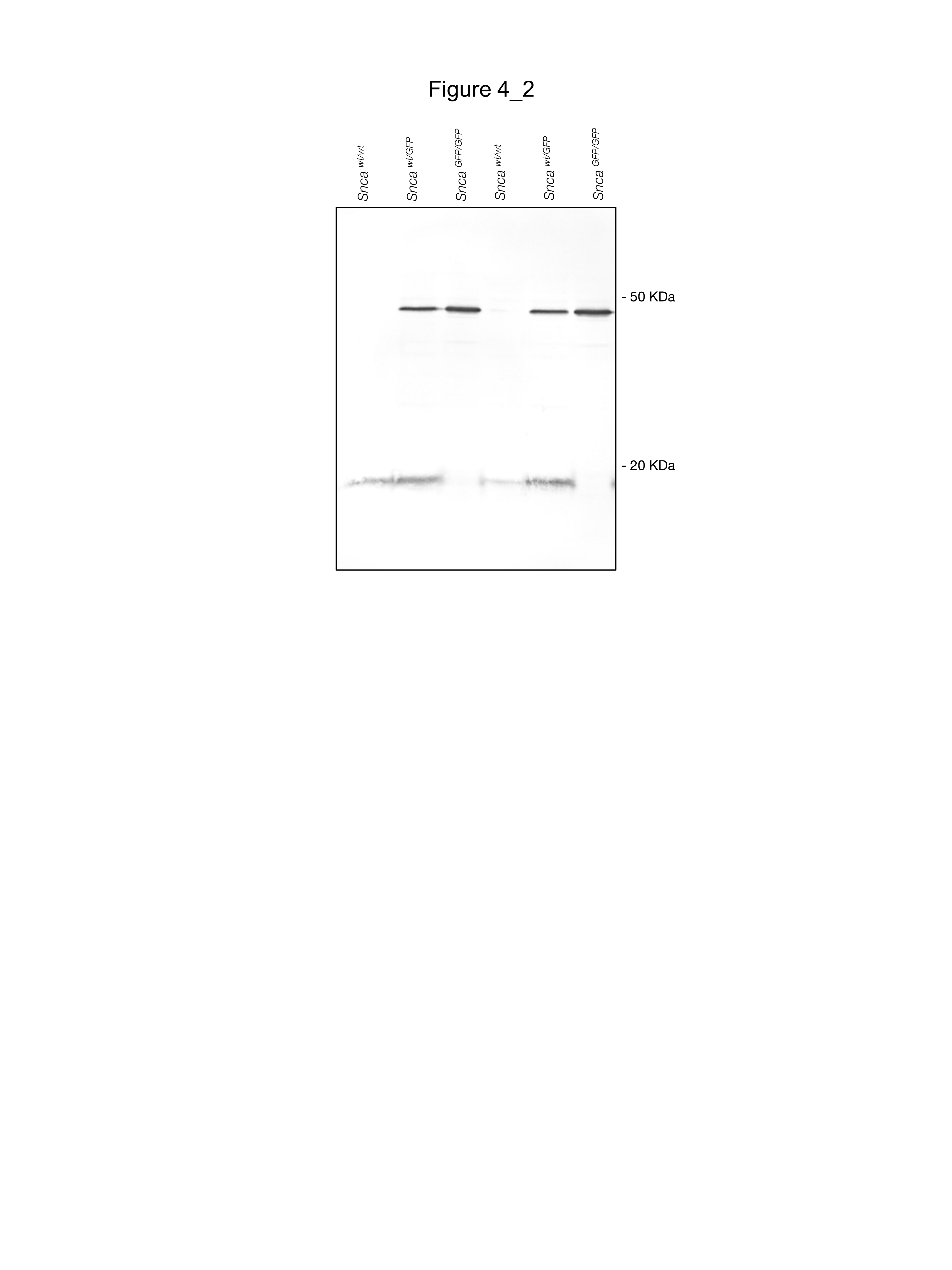

Supplement: Extended Data Figure 4-2 — Full 9027 Western blotting for Figure 4C. Download Figure 4-2, TIF file. [file enu-eN-MNT-0007-20-s05.tif]

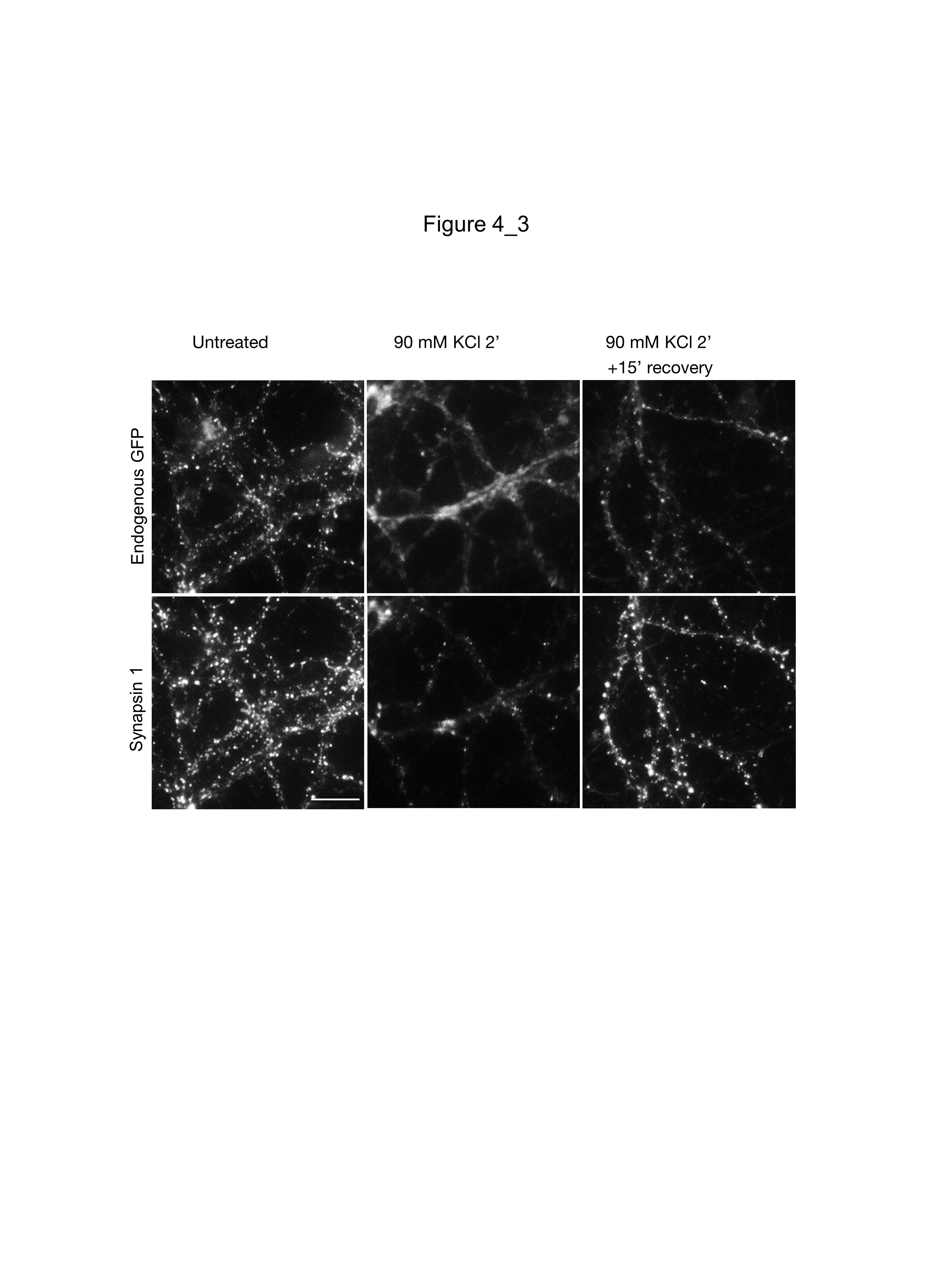

Supplement: Extended Data Figure 4-3 — Sncawt/GFP neurons were fixed before, after a 2-min exposure to 90 mm KCl, or after a 15-min recovery from HK stimulation and co-stained with an anti-Synapsin 1 antibody. Scale bar: 10 µm. Download Figure 4-3, TIF file. [file enu-eN-MNT-0007-20-s06.tif]

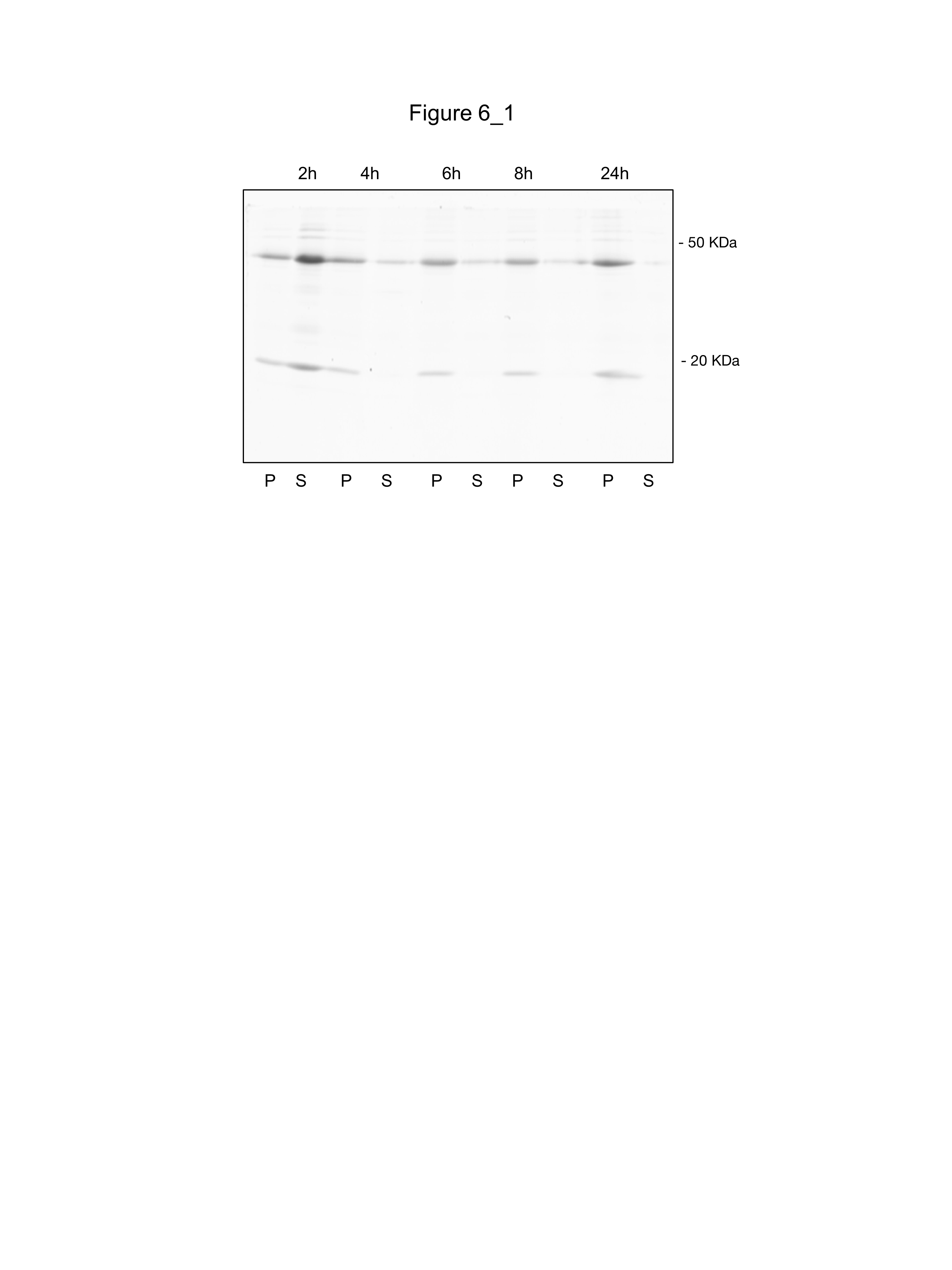

Supplement: Extended Data Figure 6-1 — Full Western blottings (Syn9027) for Figure 6A, right panels. Download Figure 6-1, TIF file. [file enu-eN-MNT-0007-20-s07.tif]

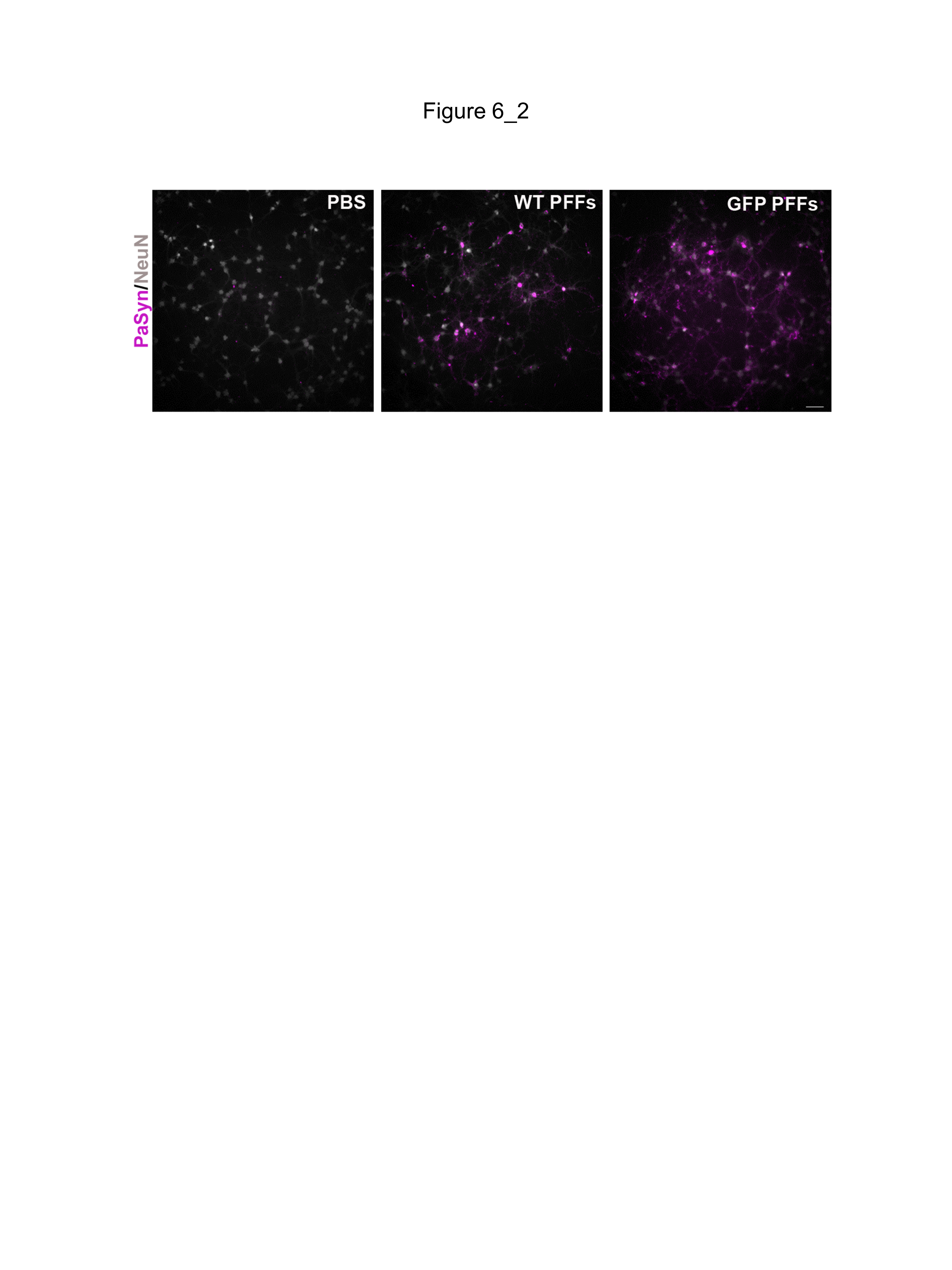

Supplement: Extended Data Figure 6-2 — Wt (CD1) primary hippocampal neurons were treated with wt or GFP-tagged aSyn PFFs and stained for phosphorylated aSyn (pSyn) and NeuN 14 d post-PFFs addition. The presence of pSyn signal in aSyn-GFP PFFs-treated neurons demonstrate that these fibrils are competent in inducing aSyn pathology. Scale bar: 20 µm. Download Figure 6-2, TIF file. [file enu-eN-MNT-0007-20-s08.tif]

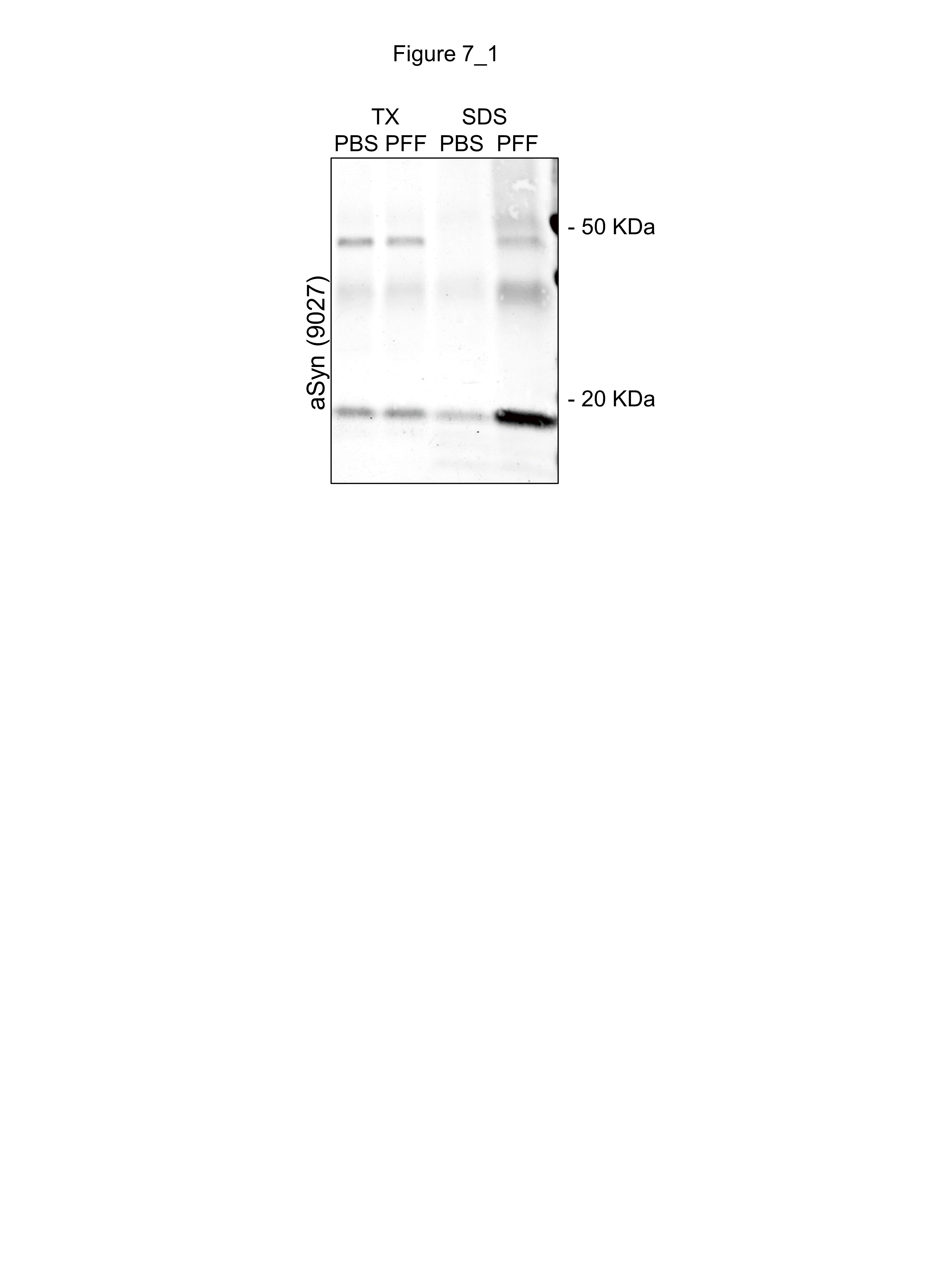

Supplement: Extended Data Figure 7-1 — Additional Western blotting (Syn9027) of Sncawt/GFP neurons treated for 14 d with PBS or PFFs and sequentially extracted with 1% TX-100 followed by 2% SDS. Download Figure 7-1, TIF file. [file enu-eN-MNT-0007-20-s09.tif]

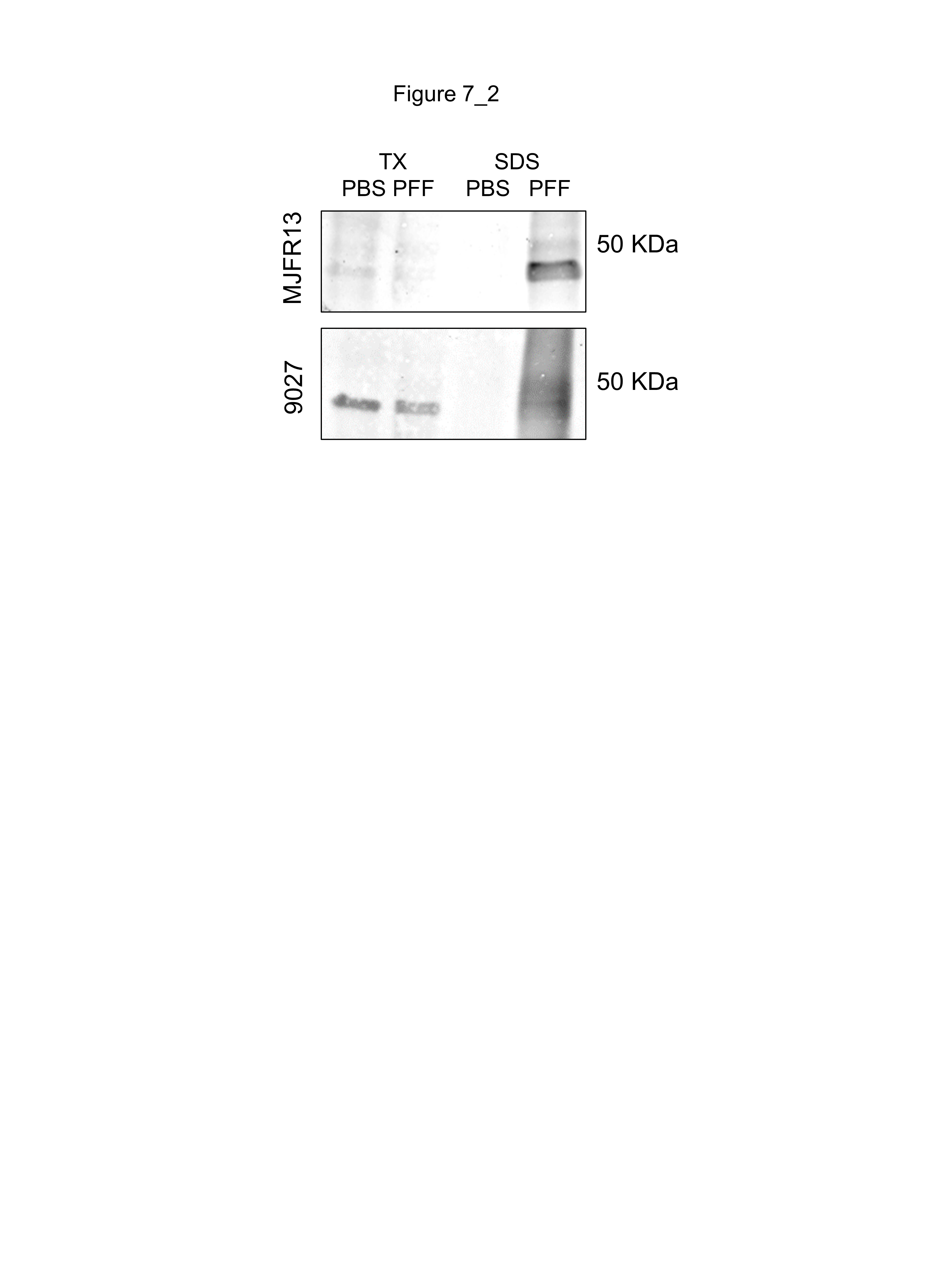

Supplement: Extended Data Figure 7-2 — Western blottings of primary Sncawt/GFP hippocampal neurons following exposure to PBS or PFFs and sequential extraction with 1% TX-100 (TX) and 2% SDS. Phosphorylation of aSyn-GFP insoluble aggregates was revealed using the MJFR13 antibody. Total aSyn was detected using the anti-aSyn antibody 9027. Download Figure 7-2, TIF file. [file enu-eN-MNT-0007-20-s10.tif]

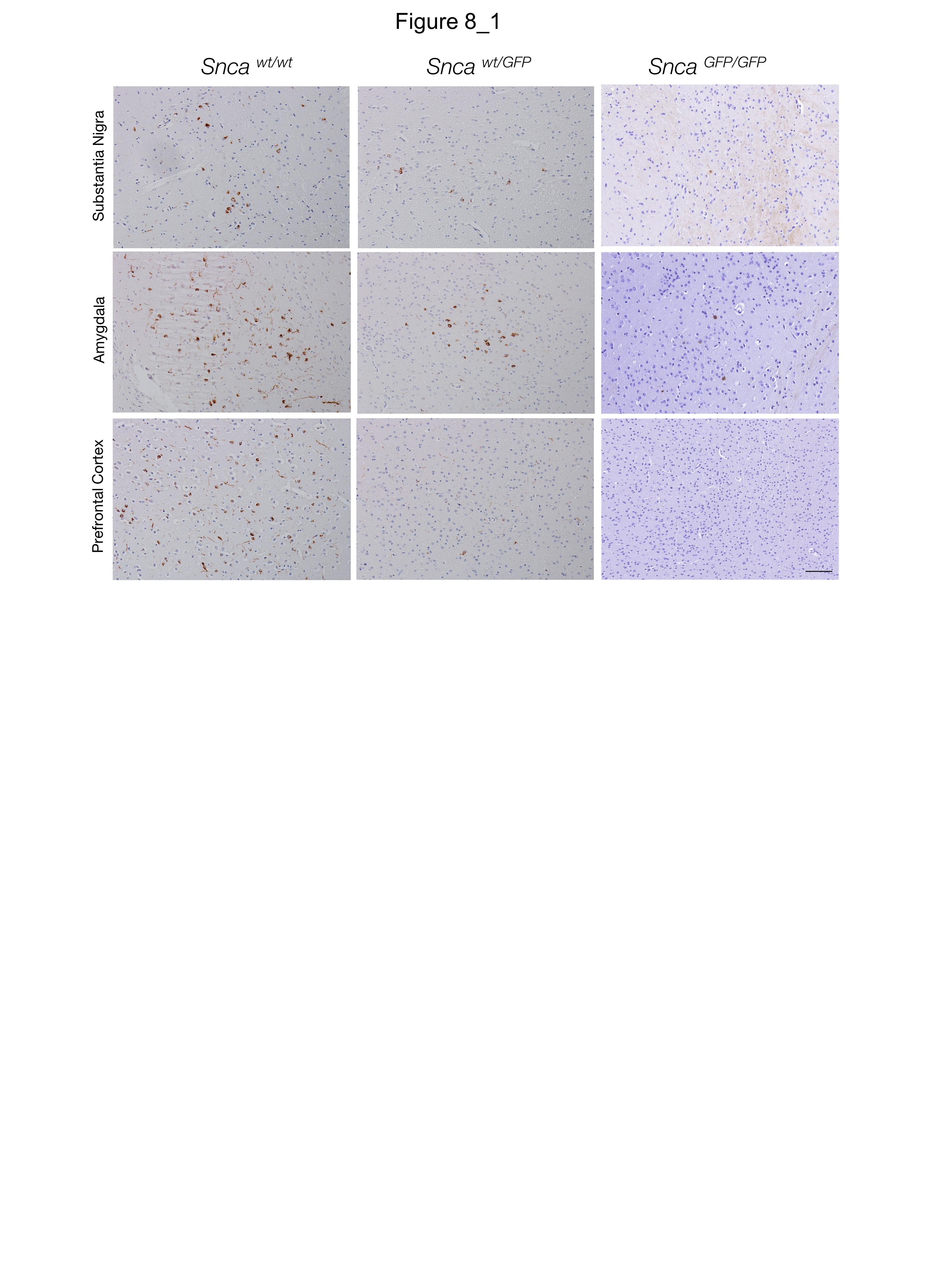

Supplement: Extended Data Figure 8-1 — Five micrograms of recombinant PFFs were injected into the dorsal striatum, ventral striatum, and cortex of wt, heterozygous, and homozygous Snca-GFP mice. Pathology was analyzed at 90 d postinjection (dpi) by immunohistochemistry using an antibody (81A) against pSyn. Scale bars: 50 µm. Download Figure 8-1, TIF file. [file enu-eN-MNT-0007-20-s11.tif]

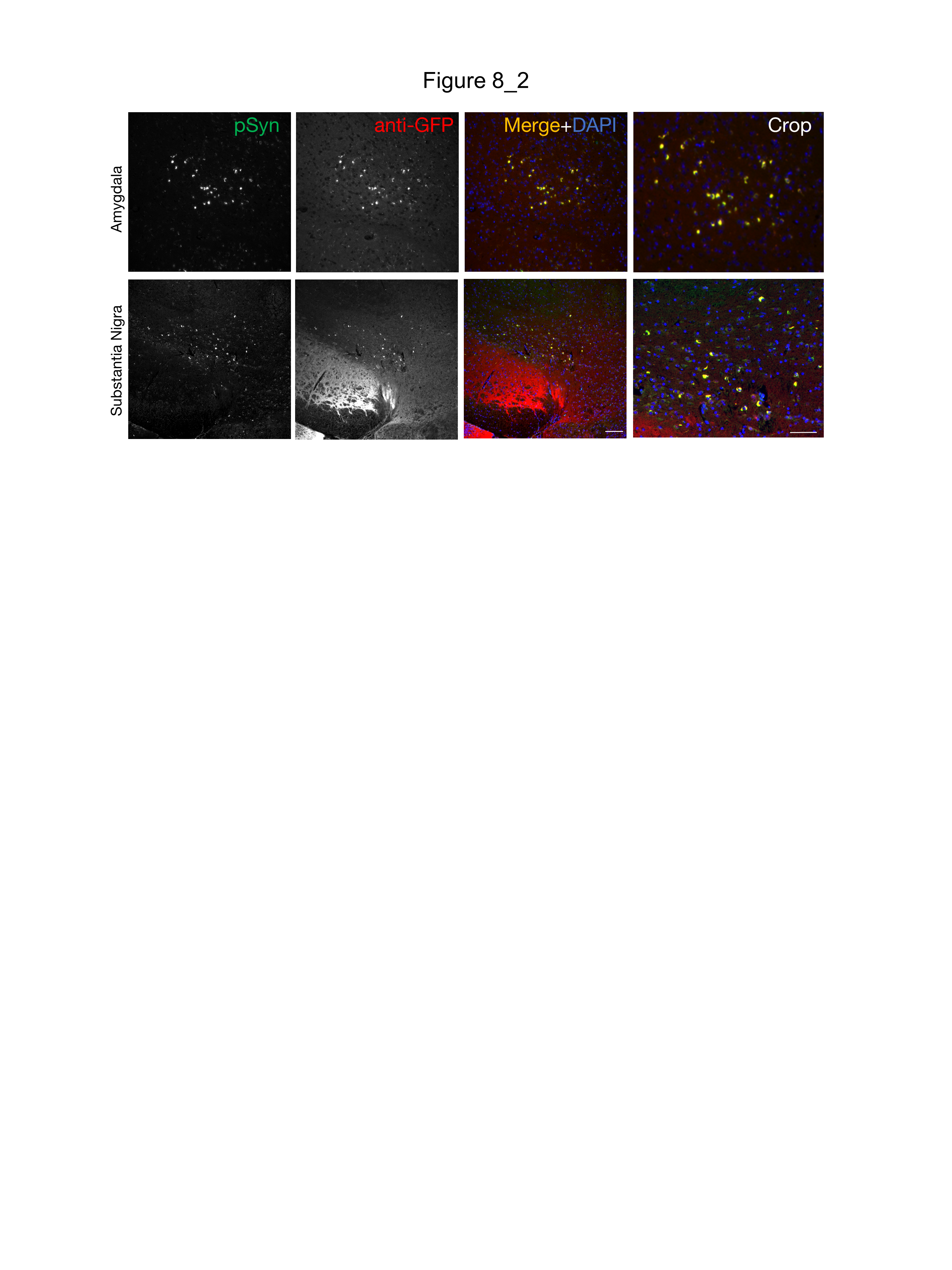

Supplement: Extended Data Figure 8-2 — Immunofluorescence using an antibody against pSyn (81A) and an anti-GFP antibody on sections of Sncawt/GFP mice injected with PFFs in the dorsal striatum, ventral striatum, and cortex show high levels of colocalization in both the substantia nigra and amygdala 90 d postinjection. Scale bars: 100 µm, 50 µm (crop). Download Figure 8-2, TIF file. [file enu-eN-MNT-0007-20-s13.tif]

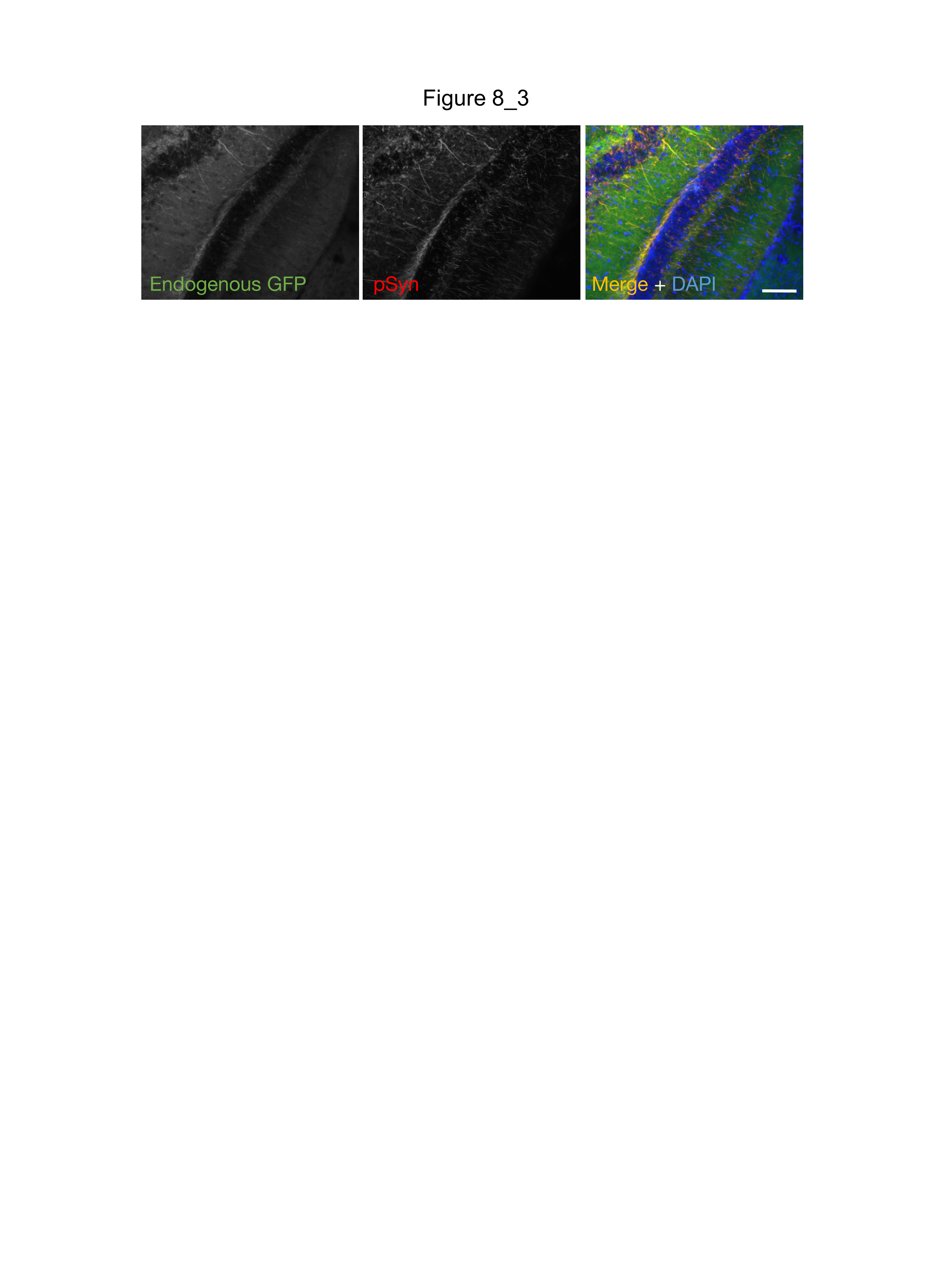

Supplement: Extended Data Figure 8-3 — Compresstome brain sections derived from Sncawt/GFP mice injected in the hippocampus with PFFs (30 d postinjection) and stained with anti-pSyn antibody (81A) show that GFP-tagged aggregates can also be detected using the endogenous fluorescence of the fluorophore. Scale bar: 50 µm. Download Figure 8-3, TIF file. [file enu-eN-MNT-0007-20-s12.tif]
